# Post‐Tuberculosis Lung Disease: Clinicopathologic Insights, Diagnosis, Prevention, and Management

**DOI:** 10.1002/mco2.70974

**Published:** 2026-09-15

**Authors:** Radha Gopalaswamy, Selvakumar Subbian

**Affiliations:** ^1^ Madurai Kamaraj University Madurai India; ^2^ Public Health Research Institute, New Jersey Medical School, Rutgers The State University of New Jersey Newark New Jersey USA

**Keywords:** antibiotics, aspergillosis, biomarkers, lung fibrosis, mycobacterium, NTM diseases, oscillometry, pulmonary function, tuberculosis

## Abstract

Post‐tuberculosis lung disease (PTLD) encompasses a spectrum of chronic pulmonary abnormalities arising from tuberculosis (TB)‐associated tissue injury and maladaptive repair, and is emerging as a major contributor to long‐term respiratory disability among TB survivors. In this review, we position PTLD within the broader continuum of TB pathobiology, from initial host–pathogen interactions to chronic structural and functional lung sequelae. We examine current definitions, epidemiology, and clinical burden, and present mechanistic insights into the inflammatory, proteolytic, fibrotic, immunometabolic, and vascular pathways that drive persistent lung damage. We further discuss the heterogeneous clinical phenotypes of PTLD, including airflow obstruction, bronchiectasis, restrictive lung disease, pleural complications, chronic pulmonary aspergillosis, and nontuberculous mycobacterial disease, highlighting practical approaches to diagnosis, risk stratification, and management across diverse healthcare settings. We discuss PTLD in children and adolescents, prevention opportunities throughout the TB care cascade, and health‐system strategies for integrating post‐TB care. Finally, we identify key knowledge gaps in PTLD nosology, biomarkers, therapeutic development, clinical trial design, and implementation science. Recognition of PTLD as a continuation rather than an endpoint of TB is essential for advancing survivorship‐focused care and reducing the global burden of chronic respiratory disease after TB.

## Graphical abstract

A comprehensive review on post‐tuberculosis lung disease (PTLD) addresses the epidemiology, pathogenesis, clinical phenotypes and management, risk factors, prevention and systems approach, and future directions. This review provides standardized definitions, mechanistic insights and biomarkers of PTLD development, and clinical strategies for PTLD management in high TB burden settings.

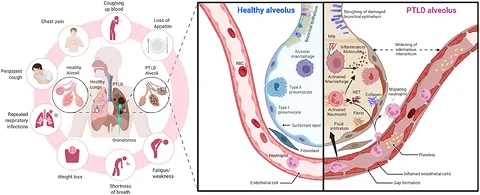

## 1 Introduction

Tuberculosis (TB), caused by *Mycobacterium tuberculosis* (Mtb), remains a major global health challenge and the leading cause of death from a single infectious agent, surpassing HIV/AIDS and malaria [1]. In 2024, an estimated 10.7 million new cases and 1.23 million deaths were reported worldwide, underscoring the persistent burden of TB despite decades of control efforts [1]. While treatment for drug‐susceptible TB achieves about 88% cure rates, the emergence of multidrug‐resistant TB (MDR‐TB) and extensively drug‐resistant TB (XDR‐TB) continues to threaten progress in TB management [2]. Importantly, even after successful microbiological cure with treatment, TB survivors face a substantial risk of developing long‐term pulmonary sequelae, collectively termed as post‐TB lung disease (PTLD), which is increasingly recognized as a major contributor to chronic respiratory morbidity, particularly in TB‐endemic countries [3, 4].

TB pathogenesis spans a dynamic spectrum, ranging from asymptomatic latent TB infection (LTBI) through incipient and subclinical stages to active disease, with each outcome defined by distinct immunopathological characteristics [2, 5, 6, 7, 8]. While global TB control measures have focused mostly on early detection and treatment of active cases and preventive therapy for LTBI, the post‐treatment phase has received very little attention. Clinically, PTLD encompasses a heterogeneous array of structural and functional abnormalities, including airflow obstruction, bronchiectasis, parenchymal fibrosis, pleural thickening, and impaired gas exchange [9, 10, 11, 12]. Epidemiological studies suggest that 10%–18% of TB survivors develop clinically significant PTLD, with higher prevalence in low‐ and middle‐income countries (LMICs) and among individuals with prior cavitary disease or delayed treatment [13, 14].

The pathobiology of PTLD reflects an interplay of persistent lung inflammation, protease‐driven tissue destruction, and dysregulated tissue repair, compounded by immunometabolic shifts and fibrogenic signaling at the cellular level [15, 16]. These processes not only impair lung function but also predispose to secondary infections such as chronic pulmonary aspergillosis (CPA) and nontuberculous mycobacterial (NTM) disease [14, 15, 17, 18, 19, 20, 21, 22]. Thus, recognizing PTLD as a distinct clinical entity is essential for developing new and/or improved diagnostics, preventive strategies, and host‐directed interventions to mitigate its long‐term impact on global respiratory health [23, 24]. Despite its high prevalence and impact, PTLD is largely ignored in national and international TB program guidelines, including routine post‐treatment follow‐up strategies [25, 26]. Most TB programs declare patients “successfully treated” at treatment completion without structured assessment for downstream lung disease. Similarly, several uncertainties remain regarding risk stratification, optimal screening strategies, preventive interventions during TB treatment, and cost‐effective models of care for PTLD [24, 25, 27]. Therefore, review on PTLD is urgently needed to discuss the etiology, disease burden, policy neglect, and fragmented evidence base surrounding this condition, as well as to harmonize terminology, compare methodological approaches, and identify reproducible outcome metrics. Such a review would not only provide emerging clinical and epidemiological data but also help shift TB care paradigms toward long‐term survivorship and chronic disease management, with significant implications for TB treatment outcomes and global health policy.

In this review, we discuss the spectrum of TB to provide the context for PTLD, discuss epidemiology, pathogenic mechanisms, clinical phenotypes, diagnosis, and management of PTLD. We also elaborate integrated diagnosis for efficient management principles across PTLD phenotypes, focusing on prevention and health‐system approaches. Finally, we discuss the challenges and knowledge gaps in PTLD management.

## 2 The TB Spectrum: From Mtb Exposure to Sequelae

**FIGURE 1:**
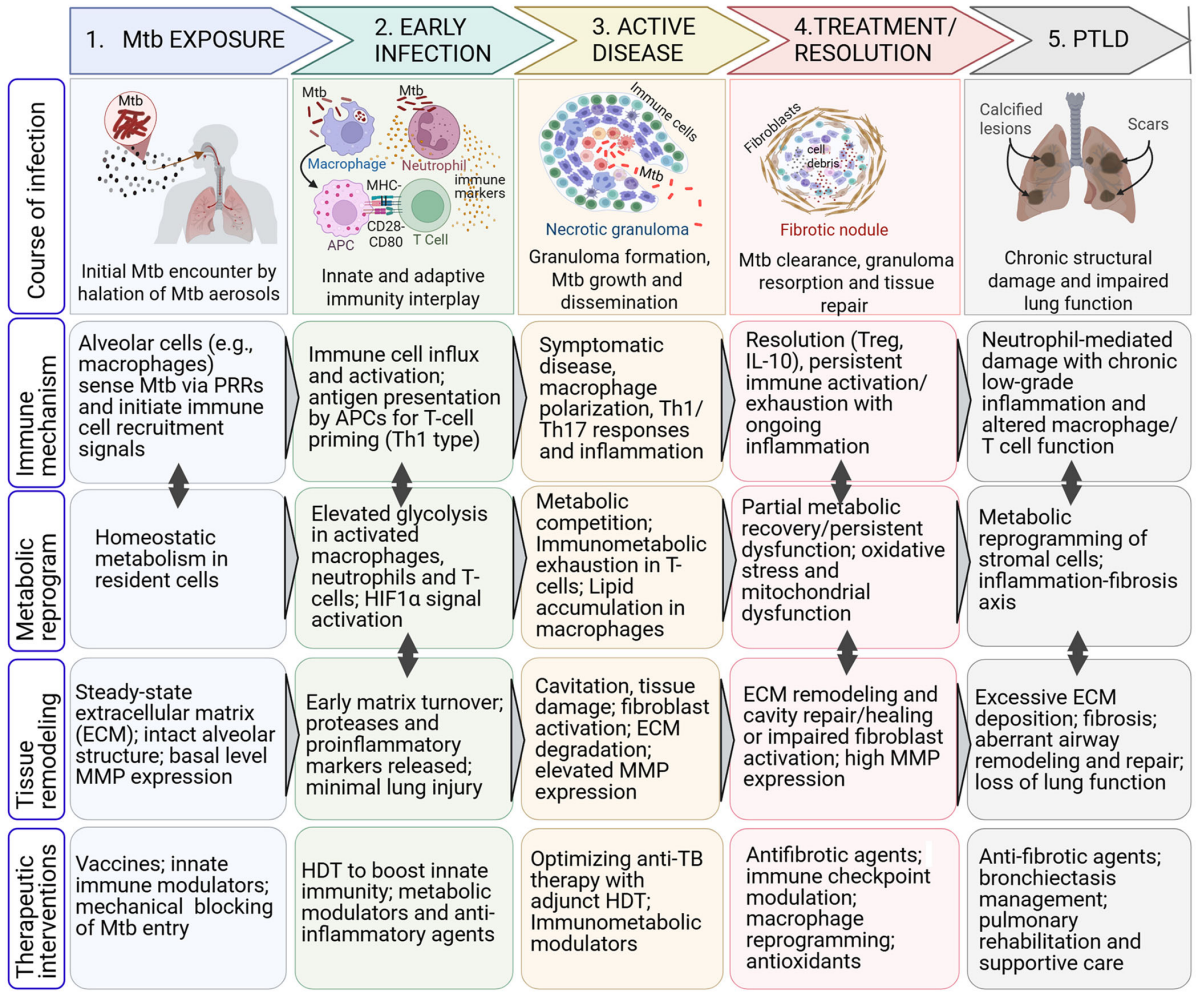
Overview of mechanistic continuum from Mtb exposure to PTLD development. The top panel indicates the kinetics of progressive Mtb infection in the lungs, from exposure to early stages of infection, symptomatic active disease, and treatment to post‐treatment PTLD sequelae. The dynamic interplay between immune dysregulation, immunometabolic reprogramming, tissue remodeling, and structural lung damage across different stages of infection are shown within the boxes. Key cellular players, including macrophages, neutrophils, T cells, and fibroblasts, play critical and divergent roles from early stages of infection to the onset of PTLD. Host factors activated by Mtb infection, and subsequently by inflammation, including drivers of cellular and tissue injury, immunometabolism, fibrogenic processes as well as vascular and pleural changes associated with PTLD are summarized in the corresponding boxes. In a subset of TB survivors who completed anti‐TB therapy, the PTLD symptoms are resolved, showing minimal disease manifestations. However, many TB survivors have lung fibrosis and structural modifications, such as scar formation, which impact the quality of life and increase the risk for subsequent infection with Mtb and/or any infectious agents. Potential therapeutic interventions to curtain PTLD, including vaccination and host‐directed therapy (HDT) should start from early stages of Mtb infection. During active TB, HDTs can be administered as adjunct to standard antibiotic therapy for TB. The figure was created with BioRender.com. APC, antigen presenting cells; ECM, extracellular matrix; HDT, host‐directed therapy; HIF‐1α, hypoxia inducible factor‐1 alpha; IL, interleukin; MMP, matrix metalloproteinases; Mtb, *Mycobacterium tuberculosis*; PRRs, pattern recognition receptors; TB, tuberculosis; Th1, T‐helper‐1 type; Treg, regulatory T cells.

TB transmission occurs primarily through inhalation of aerosolized, Mtb‐containing droplet nuclei of 1–5 µm, which are expelled when patients with pulmonary TB cough, sneeze, or speak [7, 28]. Following exposure, approximately 20%–25% of individuals become infected with Mtb, while only 5%–10% progress to active TB disease within the first 2 years of exposure. The remainder individuals develop LTBI, characterized by immunologic evidence of Mtb infection without clinical or radiologic disease [2, 7]. However, individuals with LTBI can reactivate as symptomatic active TB upon immunosuppressing host conditions [2, 7]. LTBI is typically detected by tuberculin skin testing or interferon‐gamma release assays, reflecting the recognition of Mtb antigens by the host adaptive immunity [2]. However, the TB spectrum is increasingly recognized as a dynamic continuum, rather than a binary LTBI or active disease dichotomy (Figure 1 and Table 1) [28, 29]. Accordingly, incipient TB represents an early stage of infection in which viable bacilli persist with intermittent replication, often detectable only through molecular techniques or transcriptomic biomarkers [30]. Similarly, subclinical TB is defined by metabolically active bacilli and the presence of radiologic or pathologic abnormalities in the absence of overt clinical symptoms. However, individuals with subclinical disease can be infectious and contribute to community transmission, underscoring the limitations of symptom‐based screening [28, 29, 30, 31]. Active TB, the most clinically apparent stage, manifests as cough, fever, weight loss, and night sweats, and is confirmed by microbiological evidence for Mtb, such as smear microscopy, culture, or nucleic acid amplification tests [32]. Mechanistically, the progression of Mtb infection to symptomatic disease along this spectrum is governed by a complex interplay of host immunity, pathogen burden, and environmental factors [33, 34, 35]. Effective containment of Mtb relies on robust innate and adaptive responses, including macrophage activation, Th1 cytokine signaling, and granuloma formation [7, 34, 36, 37, 38]. However, granulomas exhibit spatial and temporal heterogeneity, with regions of hypoxic, necrotic cores, and variable immune cell composition that influence bacillary persistence and treatment response [2, 39, 40]. Disruption of this equilibrium, either by immunosuppression, malnutrition, or coinfections, can precipitate reactivation and progression to active disease [28].

**TABLE 1:** Summary of the TB spectrum and key features.

Spectrum stage	Clinical features	Imaging	Microbiology	Immunologic/metabolic hallmarks	References
LTBI	Asymptomatic	Usually normal	Negative	TST/IGRA positive; controlled viable bacilli	[2, 7]
Incipient TB	Asymptomatic	Often normal/subtle	Often negative	Viable bacilli with intermittent replication; biomarker‐defined	[2, 7, 30]
Subclinical TB	Minimal symptoms	Radiological abnormalities common	Sputum/molecular positivity possible	Metabolically active, potentially infectious	[28, 29, 30, 31]
Active TB	Cough, fever, weight loss, sweats	CXR/CT with consolidation, cavities	Smear/culture/NAAT positive	High inflammatory tone; tissue injury programs	[2, 7, 32]
PTLD	Variable: cough, dyspnea, wheeze, hemoptysis	Bronchiectasis, fibrosis, cavities, pleural scarring	Usually negative for Mtb	Protease‐dominant injury, fibrosis, immunometabolic shifts	[4, 11, 12]

Abbreviations: CT, computed tomography; CxR, chest X‐ray; IGRA, interferon‐gamma release assay; LTBI, latent TB infection; NAAT, nucleic acid amplification test; PTLD, post‐TB lung disease; TB, tuberculosis; TST, tuberculin skin test.

In summary, the recognition of TB as a “life‐course” disease, ranging from exposure through post‐treatment sequelae, has profound implications for public health strategies. TB‐associated lung diseases (TALDs) may initiate before or during active TB as respiratory abnormalities and persist after the completion of TB treatment. TALD is associated with direct infection, inflammation and lung damage, though it could be asymptomatic or symptomatic. PTLD is a TALD that specifically refers to structural and functional pulmonary abnormalities following microbiological cure of a previous active TB disease [41]. Therefore, interventions to curtail TB must extend beyond bacteriologic cure to encompass early detection of subclinical disease, prevention of structural damage, and systematic post‐treatment surveillance to mitigate the long‐term burden of PTLD [31, 42]. Among the sustainable development goals of the END‐TB strategy, the catastrophic cost includes prevention, cure, rehabilitation, and long‐term care emplacing the importance of TALD post‐treatment [1].

## 3 Definition and Epidemiology of PTLD

The TB spectrum does not end with microbiological cure of symptomatic disease upon anti‐TB treatment. Rather, PTLD ensues as the sequelae of progressive Mtb infection and post‐treatment, encompassing structural and functional abnormalities that persist or evolve after therapy [3, 4, 18, 43, 44].

### 3.1 Definition of PTLD

**FIGURE 2:**
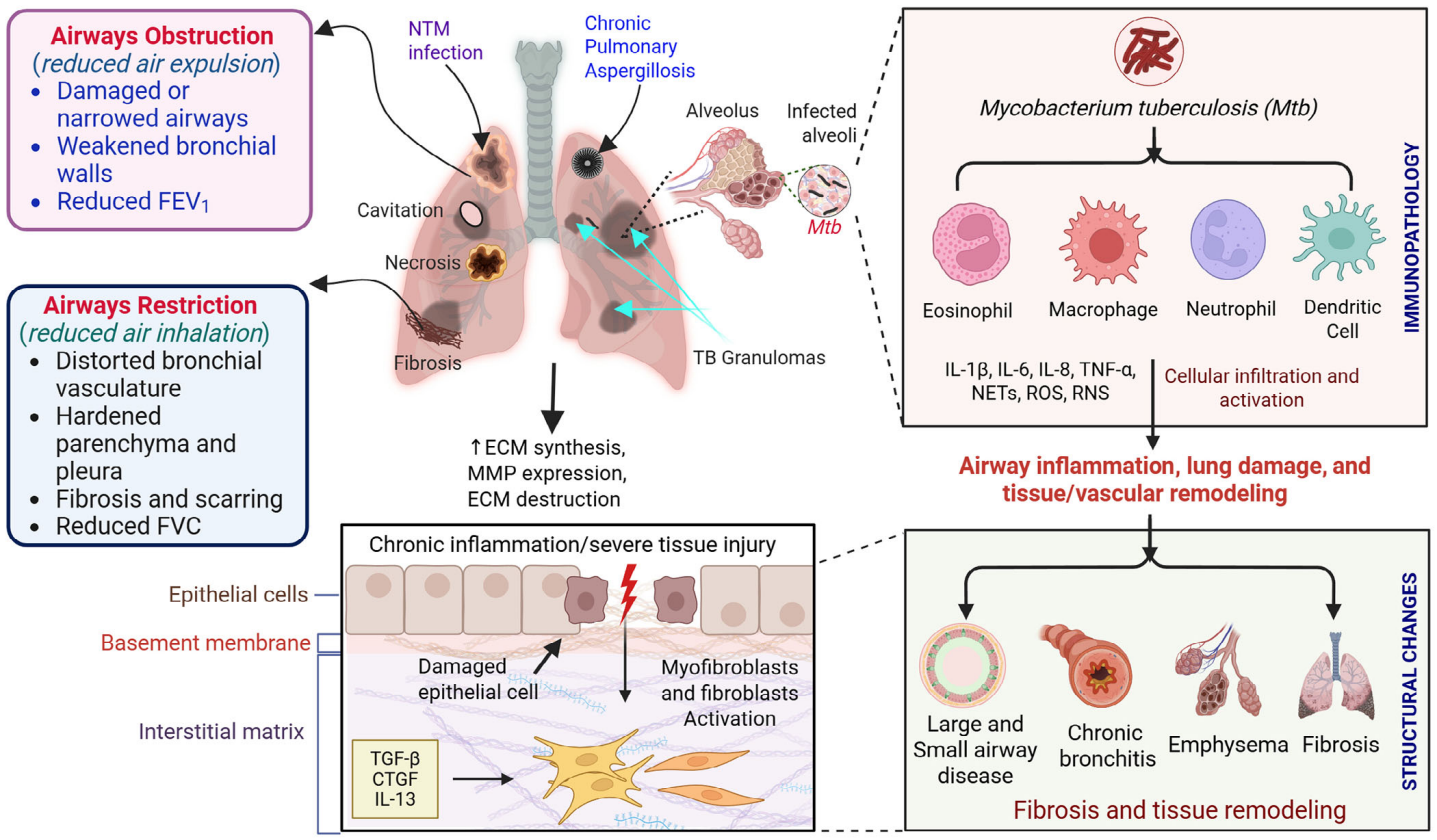
Cellular and molecular landscape of PTLD development. A schematic representation of the lung microenvironment following Mtb infection, highlighting the interplay between immune and nonimmune cells, molecular mediators, and pathological outcomes. Necrotic and cavitary granulomas, NTM infection, CPA, fibrotic scars, and vascular remodeling are key drivers and components of PTLD. Airway obstruction and restriction, as well as fibrosis and scarring are the primary PTLD manifestations. At the alveoli/TB granuloma interface, Mtb‐infected macrophages, dendritic cells and neutrophils, as well as eosinophils release proinflammatory cytokines, reactive oxygen and nitrogen species (ROS and RNS), which amplify airway inflammation, leading to cavitation, large and small airway disease, chronic bronchitis, emphysema and fibrosis. Chronic inflammation and tissue destruction also leads to activation of fibroblasts, causing elevated extracellular matrix (ECM) and matrix metalloproteinases (MMP) expression, leading to matrix degradation. The figure was created with BioRender.com. CTGF, connective tissue growth factor; ECM, extracellular matrix; FEV, forced expiratory volume; FVC, forced vital capacity; IL, interleukin; MMP, matrix metalloproteinases; NET, neutrophil extracellular traps; NTM, nontuberculous mycobacterium; PTLD, post‐TB lung disease; RNS, reactive nitrogen species; ROS, reactive oxygen species; TGF, transforming growth factor; TNF‐α, tumor necrosis factor‐alpha.

PTLD may be defined as evidence of chronic pulmonary abnormality attributable to previous TB, with or without current respiratory symptoms and irrespective of anti‐TB treatment outcome [3, 4]. This definition encompasses a wide spectrum of structural and functional sequelae in the lungs, including physiological impairments ranging from airflow obstruction to restrictive defects, mixed ventilatory patterns, reduced diffusing capacity and impaired gas exchange (Figure 2). These pulmonary function impairments are accompanied by radiologic abnormalities such as bronchiectasis, fibrotic bands, pleural thickening, and residual granulomatous cavities in the infected lungs [9, 10, 11, 12]. Importantly, PTLD is not a single entity but a heterogeneous syndrome reflecting the cumulative impact of lung destruction during active disease and maladaptive repair processes after microbiological cure with antibiotic treatment [15, 16]. The clinical spectrum of PTLD includes previous history of microbiologically cured TB, symptoms, imaging indicating structural damage, and spirometry or other tests indicative of functional abnormalities [9, 18, 24, 45, 46]. Considering this evidence, the case definition for PTLD includes three components—a previous history of TB showing pulmonary or pleural involvement, with no clinical disease during evaluation; pulmonary abnormalities in more than two clinical domains indicated as abnormal chest imaging, impaired lung function or respiratory symptoms; clear association of the clinical physiology with a previous episode of TB disease [9].

### 3.2 Epidemiology of PTLD

Recent epidemiological estimates indicate that PTLD affects approximately 10%–18% of individuals treated for pulmonary TB, though prevalence varies by population, diagnostic criteria, and follow‐up duration [4, 13, 14]. In longitudinal cohort studies, clinically significant impairment has been documented in TB patients from as early as 1 year to more than three decades post‐treatment, with a median onset between 5 and 11 years [4]. The persistence of chronic respiratory symptoms in PTLD contributes 42%–47% of total disease burden. Out of 122 million disability‐adjusted life years (DALYs) globally due to TB, 58 million were contributed by post‐TB sequelae. Although the burden of post‐TB sequelae was distributed over survival years, one third of the DALYs gradually increases within 15 years or more post‐TB treatment [41, 47]. The burden is disproportionately higher in TB‐endemic LMICs, where delayed diagnosis, limited access to imaging, and higher rates of severe or DR‐TB amplify the risk of residual lung damage [48]. However, emerging data from high‐income settings suggest that PTLD is under‐recognized globally, even where TB incidence is low [14].

In addition, risk stratification studies have highlighted several determinants of PTLD in patients, including the extent of initial disease, presence of cavitation, smear positivity, and treatment delays [13, 49]. Importantly, DR‐TB, particularly MDR‐TB, was strongly associated with mixed obstructive and restrictive patterns with more severe radiologic sequelae, which suggest prolonged bacterial persistence and cumulative inflammatory injury in the lungs [13]. Additional aggravating and/or contributing factors of PTLD include older age, smoking, alcohol use, malnutrition, and comorbidities such as HIV infection, though the latter shows variable associations across clinical and epidemiological studies [48, 50, 51]. While a majority of the cases with drug‐susceptible TB showed predominantly a restrictive pulmonary disorder, those with MDR‐TB had a combination of restrictive and obstructive lung impairment [13]. Typically, the forced expiratory volume in 1 min (FEV_1_) of the lung was affected in most PTLD cases over their forced vital capacity (FVC) [14].

While the morbidity associated with PTLD is assessed by different studies, the data on mortality calculation due to exclusive PTLD are lacking. Previous studies indicated 15%–28% death post‐TB treatment when followed across 10 years losing a median life age of 23.8 years. The cause of death includes pulmonary abnormalities including asthma, chronic obstructive pulmonary disorder (COPD), cancer; myocardial infarction; hypertension; renal disease; malnutrition; influenza pneumonia; cerebrovascular diseases; and post‐TB sequelae [52, 53, 54, 55]. A recent study used mathematical modeling to estimate the life years lost and quality‐adjusted‐life‐years (QALY) for post‐TB sequelae patients compared to no‐TB control cohort. Mortality due to co‐prevalent conditions was estimated additively with incremental difference due to post‐TB sequelae established. The mean life lost years were 41% and 48% QALY with median age of 65–74 years and predominantly men [56, 57]. Further studies on mortality due to PTLD are warranted that would enable the release of global guidelines for recommendations to PTLD management.

In summary, despite its clinical significance and importance, mitigating PTLD has been neglected from traditional TB control frameworks, which narrowly emphasize bacteriologic cure as the endpoint of care [58, 59]. This gap in TB management measures has major implications for global health, as PTLD contributes to chronic respiratory disability, reduced quality of life, and increased susceptibility to secondary infections such as CPA and NTM diseases [58, 59]. Therefore, standardized definitions of PTLD manifestations and systematic post‐treatment surveillance of TB cases, as well as the integration of pulmonary function testing into TB eradication programs are urgently needed to quantify the true burden of PTLD and guide interventions aimed at mitigating this long‐term sequela of TB.

## 4 Pathogenic Mechanisms of PTLD

The pathogenesis of PTLD reflects a continuum of lung injury and tissue repair processes initiated during various stages of Mtb infection and perpetuated beyond microbiologic cure through anti‐TB therapy (Figure 1) [4, 12]. Structural damage of PTLD begins early in disease evolution and is driven by the interplay between Mtb persistence, host immune responses, and the microenvironmental context of the lung (Figure 2 and Table 2) [4, 12]. The hallmark of severe pulmonary TB is cavitation, which arises from protease‐rich microenvironments that degrade the principal structural proteins of the lung, such as the elastins and collagens [11, 60, 61]. These cavities are often hypoxic and necrotic and serve as reservoirs for both dormant and actively replicating bacilli, which amplify bronchogenic spread, seeding distal airways and setting the stage for chronic airway remodeling [4, 62].

**TABLE 2:** Mechanistic axes and potential interventions for PTLD.

Axis	Pathobiology	Candidate interventions	Rationale	References
Protease/MMP	ECM degradation, cavitation, airway distortion	Doxycycline (MMP modulation), optimized antimicrobials	Reduce proteolysis, foster orderly repair	[15, 16, 63]
Fibrosis/TGF‐β‐CTGF	Myofibroblast activation, collagen deposition	Pirfenidone, nintedanib (selected cases)	Antifibrotic signaling blockade	[64, 65, 66, 67]
Oxidative/redox stress	ROS/RNS‐mediated damage, NETosis	N‐acetylcysteine, antioxidants	Restore redox balance, limit damage	[63, 68]
Immunometabolic	HIF‐1α glycolysis; succinate/itaconate	Rapamycin, Vitamin D_3_, PBA (autophagy/mTOR); PPAR/Nrf2 modulation	Rewire inflammatory metabolism; enhance bacterial clearance	[63, 69, 70]
Inflammatory cytokines	TNF‐α/IL‐1β/IL‐6‐high states	NSAIDs, cautious steroids in select contexts	Reduce injurious inflammation without impairing control	[63, 71, 72]

Abbreviations: CTGF, connective tissue growth factor; ECM, extracellular matrix; HIF, hypoxia inducible factor; IL, interleukin; MMP, matrix metalloprotease; NET, neutrophil extracellular traps; NSAIDs, nonsteroidal anti‐inflammatory drug; PBA 4‐phenylbutyrate; PPAR/Nrf2, peroxisome proliferator‐activated receptor/nuclear factor erythroid 2‐related factor 2; RNS, reactive nitrogen species; ROS, reactive oxygen species; TGF‐β, transforming growth factor β; TNF‐α, tumor necrosis factor‐alpha.

### 4.1 Role of MMPs

A central driver of tissue destruction in PTLD is the imbalance in protease–antiprotease levels, contributed by elevated expression of matrix metalloproteinases (MMPs) MMP‐1, ‐2, ‐3, ‐8, and ‐9, which degrade extracellular matrix (ECM) components and compromises alveolar integrity during Mtb infection [15, 16, 73]. While elevated MMP activities correlate with cavitation and poor treatment outcomes during TB, the tissue inhibitors of metalloproteinases (TIMPs) fail to adequately counterbalance this MMP‐mediated proteolytic surge in PTLD [16]. Furthermore, exacerbated neutrophil recruitment and activation during Mtb infection amplify this destructive cascade through neutrophil elastase release and neutrophil extracellular network formation (NETosis) [74, 75]. These processes liberate histones and proteolytic enzymes from immune cells into the extracellular milieu, exacerbating epithelial injury and fueling inflammation in the lungs [68, 76, 77]. Importantly, the NET‐associated biomarkers remain elevated in TB patients with delayed treatment response, implicating neutrophil‐driven pathology in PTLD progression [15] (Figure 2).

### 4.2 Role of Epithelial Injury

**FIGURE 3:**
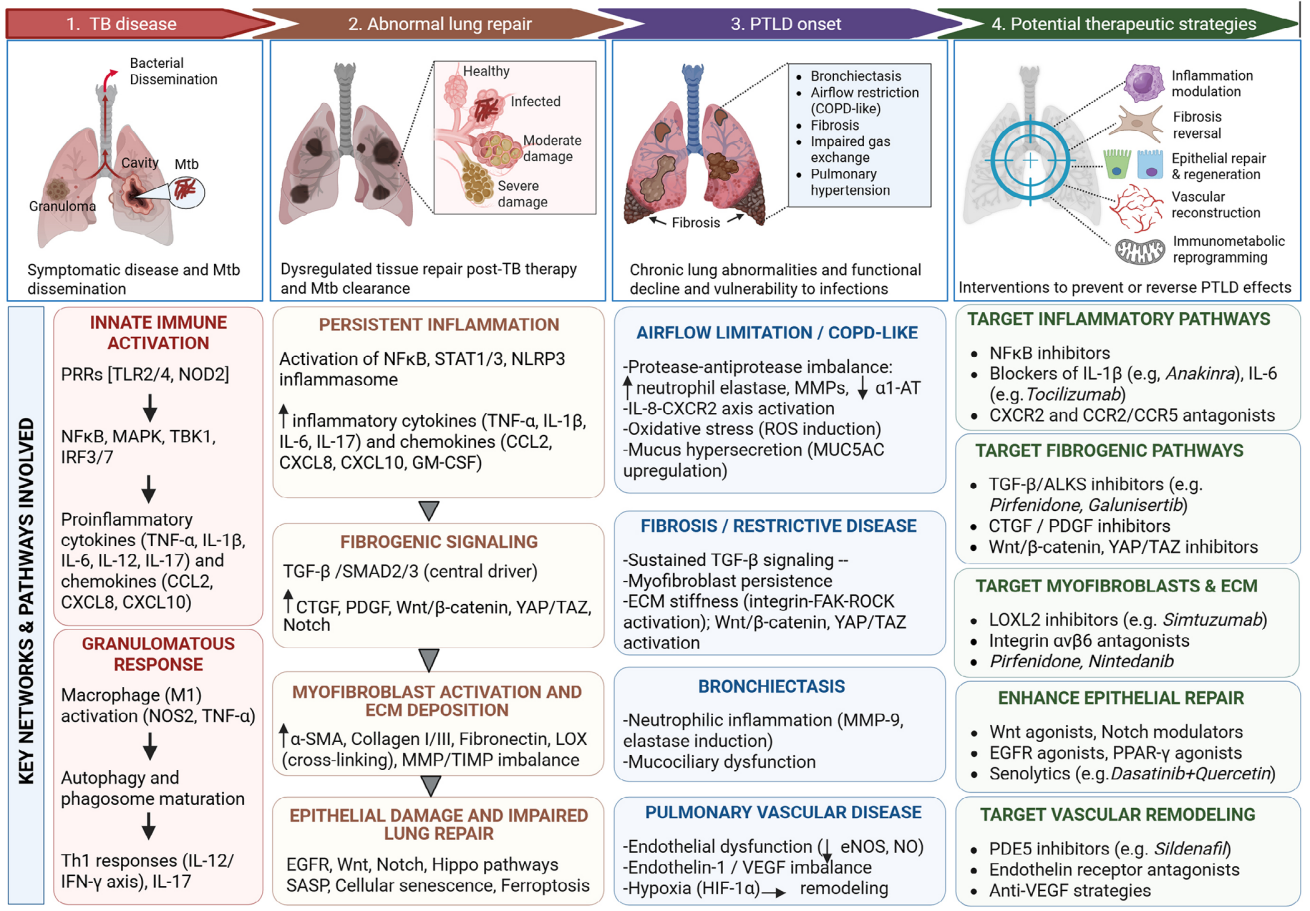
Lung fibrosis pathways and networks associated with PTLD development. Lung destruction and fibrosis during PTLD are driven by a complex interplay of cytokines, growth factors, receptors, and intracellular signaling cascades that orchestrate fibroblast activation and extracellular matrix (ECM) deposition. The upstream trigger of this cascade is innate immune activators, including proinflammatory mediators, which can result from acute lung injury, bacterial infection, or environmental insults. Infected innate cells release signals to recruit other immune cells, thus forming the granuloma, which contributes to persistent inflammation and abnormal lung repair. Bacterial infection further activates TLR signaling, enhancing NF‐κB and STAT3 pathways, which upregulate proinflammatory and profibrotic mediators. The NF‐κB/STAT‐1/3‐mediated transcriptional regulation activates the NLRP3 inflammasome and elevates inflammatory cytokines. This, together with fibrogenic signaling mediated by TGF‐β1, a master regulator of fibrosis, elevates the levels of PDGF, CTGF, Wnt/Beta‐catenin, YAP/TAZ, and Notch pathways, amplifying profibrotic signaling, and stimulating fibroblast precursors. Fibrosis response is followed by myofibroblast activation and extracellular matrix deposition, mediated by elevated levels of smooth muscle actin (SMA), collagen (COL1A1, COL3A1), fibronectin, and MMPs (MMP2, MMP9), balanced by TIMPs, balancing matrix degradation, and deposition. Excessive ECM accumulation stiffens tissue, perpetuating integrin‐mediated TGF‐β activation and sustaining fibrosis. These factors together can also lead to epithelial damage and impaired lung repair. The COPD‐like airflow limitation at the onset of PTLD is mediated by the imbalance in protease/antiprotease levels, mainly produced by activated neutrophils. Elevated elastase and MMPs with concurrent reduction of a‐AT levels results in oxidative stress and hyper mucus secretion, obstructing the airflow. Other ailments including fibrosis/restrictive disease, bronchiectasis and pulmonary vascular disease are developed, respectively, by sustained TGF‐β signaling, neutrophilic inflammation, and endothelial dysfunction, marked by the differential expression of corresponding markers. Potential treatment strategies for PTLD include targeting host pathways that regulate inflammation, fibrogenesis, myofibroblast activation, and ECM deposition as well as epithelial repair and vascular remodeling. The figure was created with BioRender.com. ALKS, alkane hydroxylase system; CCL, C–C motif chemokine ligand; COPD, chronic obstructive pulmonary disorder; CTGF, connective tissue growth factor; CXCL, C‐X‐C motif chemokine ligand; CXCR, chemokine receptor; ECM, extracellular matrix; EGFR, epidermal growth factor receptor; FAK, focal adhesion kinase; GM‐CSF, granulocyte‐macrophage colony‐stimulating factor; HIF, hypoxia inducible factor; IFN, interferon; IL, interleukin; IRF, interferon regulatory factor; LOX, lipoxygenase; LOXL, lysyl oxidase‐like; MAPK, mitogen‐activated protein kinase; MMP, matrix metalloproteinase; NF‐κB, nuclear factor kappa‐light‐chain‐enhancer of activated B cells; NLRP, NOD‐like receptor family leucine‐rich repeat containing pyrin domain protein; NOD2, nucleotide‐binding oligomerization domain‐containing protein 2; NOS, nitric oxide synthase; NOTCH, neurogenic locus notch homolog protein; PDE, phosphodiesterase; PDGF, platelet‐derived growth factor; PPAR, peroxisome proliferator‐activated receptor; PRR, pattern recognition receptor; ROCK, rho‐associated protein kinase; ROS, reactive oxygen species; SASP, senescence‐associated secretory phenotype; SMA, smooth muscle actin; SMAD, suppressor of mothers against decapentaplegic; STAT, signal transducer and activator of transcription; TAZ, transcriptional coactivator with PDZ‐binding motif; TBK, TANK‐binding kinase; TGF, transforming growth factor; TGF‐β, transforming growth factor β; TIMP, tissue inhibitor of metalloproteinase; TLR, toll‐like receptor; TNF, tumor necrosis factor; VEGF, vascular endothelial growth factor; Wnt, wingless and Int‐1; YAP, Yes‐associated protein; α1‐AT, alpha‐1 antitrypsin.

Initial epithelial injury in PTLD, either due to direct bacterial infection and cytotoxicity, immune‐mediated damage, or environmental insults, leads to the release of host damage‐associated molecular patterns (DAMPs) and activation of resident immune cells. Alveolar macrophages and neutrophils respond by secreting proinflammatory cytokines such as interleukin‐1β (IL‐1β), IL‐6, tumor necrosis factor‐alpha (TNF‐α), and C–C motif chemokine ligand 2 (CCL2), which orchestrate the recruitment of monocytes, macrophages, neutrophils, and additional immune cells to the site of injury, amplifying the inflammatory response [78, 79, 80]. IL‐1β and TNF‐α activate NF‐κB signaling, promoting fibroblast activation and ECM deposition, while IL‐6 sustains chronic inflammation through signal transducer and activator of transcription‐3 (STAT3) signaling [81, 82, 83, 84]. Notably, CCL2/CC chemokine receptor 2 (CCR2) signaling plays a pivotal role in monocyte chemotaxis and fibroblast activation, contributing to fibrosis across multiple organ systems including the lungs [85, 86, 87]. These cytokines also stimulate the production of transforming growth factor‐beta (TGF‐β1), platelet‐derived growth factor (PDGF), vascular endothelial growth factor (VEGF), and connective tissue growth factor (CTGF), which are central to the initiation and amplification of fibrotic signaling [81] (Figure 3). The persistent inflammatory milieu sets the stage for chronic fibroblast activation and ECM remodeling.

### 4.3 Role of Fibrosis

When the bacterial burden declines in the lungs either due to host immune response or antibiotic treatment, the immunologic landscape shifts toward a fibrogenic remodeling [15, 65, 70]. Particularly, the persistent activation of host TGF‐β/suppressor of mothers against decapentaplegic (SMAD) signaling and CTGF pathways drives the fibroblast‐to‐myofibroblast differentiation and excessive collagen deposition as well as scar maturation in PTLD [15, 65, 70]. Indeed, the fibrotic lung granulomas are enriched with anti‐inflammatory, Th2 and regulatory T cells, suggesting a profibrotic cytokine milieu during tissue repair in Mtb‐infected lungs [88, 89]. Similarly, the crosstalk between wingless and Int (Wnt)/β catenin and Yes‐associated protein (YAP)/transcriptional coactivator with PDZ‐binding motif (TAZ) pathways act as mechanotransducers and stiffens the ECM, entrenching irreversible lung fibrosis, while generating traction‐bronchiectasis and pleural thickening [15, 70].

### 4.4 Role of Host Cell Cytokine and Metabolic Pathways

In addition to the structural components, the local cytokine networks activated in the lungs impact the tissue repair during TB (Table 2). While proinflammatory TNF‐α is crucial for granuloma formation and integrity, excessive TNF‐α contributes to cellular necrosis and tissue injury [90]. Similarly, IL‐1β promotes neutrophil recruitment and MMP induction, whereas elevated IL‐6, IL‐8, and IL‐12 correlate with cavitation and bronchial thickening during TB [11, 70]. These inflammatory circuits are tightly coupled to immunometabolic rewiring of corresponding host cells [91, 92]. For example, hypoxia and nutrient limitation stabilize a master transcriptional regulator, HIF‐1α, which switches the macrophage metabolism toward glycolysis, and sustains the proinflammatory response of immune cells during Mtb infection [91, 93]. Similarly, accumulation of succinate during Mtb infection amplifies IL‐1β production, while itaconate exerts counter‐regulatory effects via Nrf2 activation [15, 68, 70]. In addition, lipid mediator balance, particularly the ratio of prostaglandin E_2_ to lipoxin A_4_, further modulates the granulomatous lesion's fate, influencing whether repair culminates in resolution or fibrosis in the lungs [70] (Figures 1 and 2).

### 4.5 Role of Cellular Senescence and Persistent Bacterial Presence

In PTLD, resolution of fibrosis is often impaired due to persistent infection, oxidative stress, and cellular senescence. NADPH oxidase 4 (NOX4) generates reactive oxygen species (ROS), inducing senescence in fibroblasts and epithelial cells [94, 95]. Senescent cells express p16 and p21, contributing to a senescence‐associated secretory phenotype (SASP) that perpetuates inflammation and fibrosis, locking the lung in a chronic fibrotic state [96, 97, 98]. This maladaptive response locks the lung in a chronic fibrotic state, resistant to repair and regeneration (Figure 3).

### 4.6 Role of Vascular and Pleural Compartments

Finally, the vascular and pleural compartments are also involved in the PTLD manifestations [4, 99]. For example, the pulmonary vascular rarefaction and remodeling contribute to pulmonary hypertension and impaired gas exchange, while pleural fibrocalcific scarring restricts the chest wall compliance and reduces diffusing capacity measured as diffusion capacity of the lungs for carbon monoxide (DLCO); these alterations can lead to the functional limitation of the lungs during PTLD [61, 100, 101]. In summary, the interlocking of various pathways, including the protease‐driven destruction, fibrogenic signaling, and metabolic reprogramming define the mechanistic architecture and function of lungs during TB and highlight potential therapeutic targets for host‐directed interventions for PTLD (Figure 1).

## 5 Clinical Phenotypes, Diagnostics, and Management of PTLD

### 5.1 Clinical Presentation and Diagnosis of PTLD

PTLD includes a variety of clinical indications depending on the extent of lung damage following TB treatment (Table 3). It ranges from asymptomatic to symptomatic conditions, which includes productive or nonproductive cough, hemoptysis, breathlessness or dyspnea, wheezing as well as lung impairment of varying degree [42, 61]. It is very critical that signs and symptoms are identified and defined, since right diagnosis of PTLD is crucial for the timely healthcare and appropriate treatment. Previous studies with longitudinal analysis of pulmonary function among TB treated cases beginning 1‐year up to 10 years across different groups indicated pronounced decline in FEV1 and FEV1/FVC ratio. One third of TB patients indicated such lung abnormalities without signs of other disorders like asthma, COPD or previous history of reduced FEV1. The post‐TB morbidity beyond cure and its progress into PTLD with debilitating lung function incurs psychosocial and economic burden requiring close follow‐up and intervention in the current health system [68, 69, 70, 71]

**TABLE 3:** Summary of clinical phenotypes, diagnosis, and management of PTLD.

Phenotype	Cardinal findings	Diagnostic tests	First‐line management	Advanced/adjunctive	References
Large airway obstruction	Stridor, exertional dyspnea; central stenosis	CT, bronchoscopy; spirometry obstruction	ICS ± SABA or LABA (if asthma‐like); LAMA (if COPD‐like); PR	Balloon dilation, stenting, ablation	[11, 12, 61, 102, 103]
Small‐airway disease	Cough, wheeze, air‐trapping	Oscillometry, plethysmography; HRCT	Bronchodilators; airway clearance; PR	Tailored anti‐inflammatory trials	[100, 104, 105]
Bronchiectasis	Productive cough, hemoptysis, exacerbation	HRCT (signet ring, lack of tapering); spirometry	Airway clearance, mucoactives, SABA/LABA/LAMA; vaccination	Inhaled antibiotics; embolization for bleeding	[3, 12, 61, 106, 107]
Parenchymal fibrosis	Dyspnea, exercise limitation, low DLCO	HRCT (fibrotic bands, traction); DLCO	PR; symptom‐guided care	Consider nintedanib/pirfenidone; oxygen for hypoxemia	[3, 12, 61, 106, 107]
Pleural disease	Pleuritic pain, restriction	HRCT; spirometry restriction	PR; analgesia	Surgical evaluation in select cases	[3, 61]
CPA	Weight loss, cough, dyspnea, hemoptysis	CT (cavity ± fungal ball), Aspergillus IgG/ELISA	Itraconazole 6–12 months; monitor levels and LFTs	Voriconazole/isavuconazole/posaconazole; amphotericin B; micafungin	[3, 58, 108, 109]
NTM disease	Chronic cough, sputum, weight loss	CT; smear/culture; NAAT	Species‐directed regimens	Multidrug ± parenteral (esp. *M. abscessus*)	[59, 110, 111]

Abbreviations: COPD, chronic obstructive pulmonary disorder; CPA, chronic pulmonary aspergillosis; CT, computed tomography; DLCO, diffusion capacity of lung for carbon monoxide; ELISA, enzyme‐linked immunosorbent assay; HRCT, high‐resolution computed tomography; ICS, inhaled corticosteroids; LABA, long‐acting beta‐agonist; LAMA, long‐acting muscarinic antagonists; LFT, liver function test; NAAT, nucleic acid amplification test; NTM, nontuberculous mycobacteria disease; PR, pulmonary rehabilitation.

**FIGURE 4:**
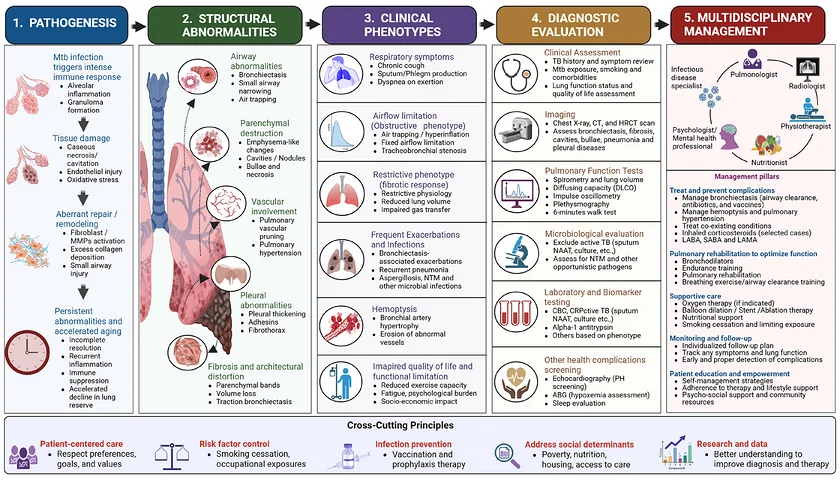
Clinical workflow for PTLD management. Linking pathogenesis, structural abnormalities, clinical phenotypes, and diagnostic evaluation are the key pillars for efficient multidisciplinary management of PTLD. Pulmonary infection by Mtb underpins the alveolar inflammation and granuloma formation that leads to tissue damage in the form of caseation, necrosis, and cavitation as well as endothelial injury. Following microbiological cure of TB, persistent inflammation, immune dysregulation, tissue destruction, and aberrant repair processes can result in chronic structural lung damage. These pathological mechanisms give rise to a spectrum of structural abnormalities, including fibrotic remodeling and architectural distortion, bronchiectasis and small‐airway disease, emphysematous and cavitary parenchymal destruction, vascular remodeling, and pleural fibrosis. Alone or in combination, these abnormalities drive distinct but often overlapping PTLD clinical phenotypes characterized by chronic respiratory symptoms, obstructive and/or restrictive physiological impairment, recurrent infective exacerbations, hemoptysis, pulmonary hypertension, reduced exercise tolerance, and impaired quality of life. Diagnostic evaluation integrates clinical assessment, thoracic imaging, pulmonary function testing, microbiological investigations, laboratory studies, and screening for cardiopulmonary complications to define disease phenotype, assess severity, exclude active or recurrent infection, and identify treatable traits. Management requires a multidisciplinary approach involving pulmonologists, infectious disease specialists, radiologists, rehabilitation specialists, mental health professionals, nutritionists, and primary care providers. Treatment is individualized according to phenotype and focuses on prevention and management of exacerbations, optimization of lung function, pulmonary rehabilitation, management of comorbidities and complications, vaccination, nutritional and psychosocial support, and long‐term monitoring. Recognition of the mechanistic links between pathogenesis, structural injury, and clinical phenotype provides the foundation for precision diagnosis, risk stratification, and comprehensive PTLD care. The figure was created with BioRender.com. ABG, arterial blood gas; CBC, complete blood count; CRP, C‐reactive protein; CT, computed tomography; DLCO, diffusing lung capacity for carbon monoxide; HRCT, high‐resolution computed tomography; LABA, long‐acting β_2_ agonists; LAMA, long‐acting muscarinic antagonists; MMP, matrix metalloproteinases; NAAT, nucleic acid amplification test; NTM, nontuberculous mycobacteria; PH, pulmonary hypertension; SABA, short‐acting beta‐agonist.

Typically, PTLD is diagnosed by chest X‐ray (CXR), computed tomography (CT), spirometry, plethysmography and impulse oscillometry (IOS), bronchoscopy and tracheoscopy for any signs, symptoms, and lung impairment associated with PTLD [112]. Treatment for PTLD is usually personalized, depending on the extent of lung impairment and associated symptoms [61, 113]. In most cases, PTLD presents as distinctive lung obstruction, restriction or both; while obstruction results in reduced lung expiration volume, restriction causes poor inhalation; each requiring a different type of treatment [9, 11, 12, 14, 18, 41, 114]. Airways‐related morbidities of PTLD can be seen in the large or small airway and could be stenotic, distorted or enlarged due to injuries to the airway walls [60, 113, 114]. Excessive inflammation and airway narrowing result in airway obstruction, causing reduced expiration of air, while excessive lung fibrosis, pleural thickening, and loss of lung parenchyma results in restrictive lung pathology, leading to reduced capacity to inhale air and increased FEV1/FVC ratio [11, 61]. The key pathological manifestations of PTLD, their diagnosis and management are discussed below and summarized in Figure 4.

### 5.2 Large and Small Airway Obstruction

Airway obstruction in PTLD spans a clinicopathologic continuum from central tracheobronchial stenosis (often postendobronchial TB) to small‐airway disease with bronchiolitis and air‐trapping [11, 12, 61]. Patients suspected of PTLD typically report stridor or exertional dyspnea, persistent cough, and sometimes hemoptysis, although these symptoms can be mistaken for asthma or chronic obstructive pulmonary disease (COPD) unless prior TB and clinical correlates are explicitly sought [11, 12, 61]. Pathologically, the chronic lung inflammation and granulomatous scarring narrow the airway lumen, while protease‐rich microenvironments and abnormal wound‐healing stiffen and distort the airway wall in PTLD [3, 12, 100]. In these cases, the high‐resolution computed tomography (HRCT) may demonstrate a concentric or eccentric stenosis, postobstructive atelectasis or air‐trapping on expiratory imaging, or a mosaic attenuation pattern from small‐airway involvement [3, 12, 100].

The spirometry analysis of PTLD cases often shows lung obstruction, marked by a reduced FEV_1_ and FEV_1_/FVC, although spirometry can be normal during early stages of the small airway disease and may fail to capture peripheral airway mechanics [104, 105]. In such cases, IOS and body plethysmography can be informative to detect increased residual volume or airway resistance [104, 105, 115, 116]. The DLCO test, which measures the lung's structural integrity, can be used for isolated airway disease associated with PTLD, although this test frequently fails when parenchymal or vascular components coexist in the lungs [100, 101].

Management of large and small airway obstruction associated with PTLD is driven by the phenotype presented in individual cases. For example, patients showing asthma‐like symptoms can be treated with inhaled corticosteroids (ICS) with or without long‐acting β_2_‐agonists (LABA), whereas those with COPD‐like symptoms may benefit from long‐acting muscarinic antagonists (LAMA) [12, 61]. The differential diagnosis of asthma and COPD over bronchiectasis, cystic fibrosis, pertussis, pneumonia, parenchymal lung disease and airway obstruction is crucial to start bronchodilators and ICS [117, 118]. Bronchodilator responsiveness (BDR) after exposure to short‐acting beta‐agonist (SABA), LABA and LAMA with or without ICS combination is recommended to confirm diagnosis of asthma or COPD. After administration of the drug, a 12% increment in FEV1 and > 200 mL in peak expiratory volume (PEV) variability over baseline was considered positive BDR and indicative of asthma or COPD to continue use of the bronchodilator agents [119, 120]. For complications such as central airway stenosis, interventional bronchoscopy can provide a durable palliation, although balloon dilation, silicone or metallic stents, and ablative modalities, such as argon plasma coagulation and electrocautery may also be used, depending on the lesion size, mucosal fragility, and presence of granulation tissue [102, 103]. In addition to these interventions, pulmonary rehabilitation (PR), including a structured aerobic and strength training with breathing retraining, should be embedded into PTLD treatment management to improve the exercise capacity and overall lung function [61, 100]. Interestingly, preventive vaccination for influenza and pneumococcal infection, as well as airway clearance techniques has been shown to reduce the risk of exacerbation of airway obstruction in patients with mucus hypersecretion or cocolonization [12, 61]. This approach may also be considered in the context of PTLD therapy.

It should be acknowledged that in the context of PTLD, a “normal” spirometry does not exclude clinically relevant small airway disease; therefore, further testing by oscillometry or plethysmography should be considered [104, 105]. Second, conditions such as wheezing and BDR can coexist with fixed stenoses and complicate PTLD diagnosis; thus, endoscopic assessment of lungs should be considered when symptoms are disproportionate to physiological findings or when localized airflow limitation is suspected on imaging [3, 12].

### 5.3 Bronchiectasis

Bronchiectasis, the permanent dilation of bronchus due to repeated infections or poor microbial clearance, is a frequent and functionally important PTLD phenotype that can occur anywhere, though it affects apical and posterior segments of the upper lobe of the lungs [3, 106]. Topographically, it often involves the apical and posterior upper lobes, which signifies as the “footprints” of prior cavitary TB, and may coexist with parenchymal scarring and traction bronchiectasis [3, 106]. While fever, fatigue, pleuritic chest pain, and dyspnea are a lesser common clinical features of bronchiectasis in PTLD cases, chronic productive cough, purulent sputum, recurrent infective exacerbations, and hemoptysis have been reported, the latter due to bronchial artery hypertrophy and fragile vasculature causing vessel rupture due to inflammation (Table 3) [12, 107]. In these cases, the diagnostic HRCT may show the hallmark “signet ring” and “lack of tapering” signs, though the sputum cultures may reveal Mtb that perpetuates inflammation and structural decline [12, 100].

Treatment of bronchiectasis in PTLD involves PR along with attempts to reduce the vicious loop of infection, inflammation and impaired airway clearance [12, 61]. In addition, since airway clearance is foundational to bronchiectasis treatment, several approaches such as oscillatory positive expiratory pressure devices, manual techniques, hydration and mucoactive agents should be considered to improve mucus transport [12, 61, 121, 122, 123]. To improve pulmonary symptoms and exercise tolerance in patients with airflow limitation or dynamic hyperinflation, LABA or LAMA may also be considered [61]. For PTLD cases with frequent bacterial infections or chronic colonization (e.g., *Pseudomonas aeruginosa*), appropriate inhaled antibiotics regimen should be considered [12, 61]. Similarly, vaccination against influenza and pneumococcus can reduce exacerbations of pulmonary dysfunction triggered by viral or bacterial pathogens coexisting with Mtb [61, 124, 125]. Interventions such as bronchial artery embolization, in‐coordination with antimicrobial and anti‐inflammatory strategies, should be considered to manage bronchiectasis in PTLD cases with massive or recurrent hemoptysis [107, 126].

Differential diagnosis of bronchiectasis in PTLD is mostly pragmatic. The traction bronchiectasis from parenchymal fibrosis may indicate tethering by scarred lung rather than recurrent infection [64, 66, 67, 100]. These patients often have fewer purulent exacerbations, and more dyspnea and reduced DLCO, and they benefit from PR, oxygen titration, and antifibrotic agents if progressive fibrosis is documented [64, 66, 67, 100]. Conversely, cylindrical or varicose bronchiectasis with frequent exacerbations demands aggressive airway clearance and prevention of exacerbation due to CPA and NTM infections, which frequently complicate bronchiectasis in PTLD cases [58, 59].

### 5.4 Parenchymal Stiffening and Pleural Complications

Parenchymal stiffening and pleural complications (PSPCs) define the restrictive end of the PTLD spectrum. In such cases, the HRCT commonly shows fibrotic bands, bronchovascular distortion, traction bronchiectasis, emphysema/gas trapping, and fibrocalcific scarring [3, 12]. In addition, pleural thickening and tethering occurs when the pleura is involved, which contributes to reduced chest wall compliance [3, 12]. Physiologically, these patients experience low DLCO, reduced FVC, exertional desaturation, and an inflated ventilatory equivalent for carbon dioxide (VE/VCO_2_) slope on cardiopulmonary exercise testing, due to reduced pulmonary vascular volume, impaired gas exchange, and ventilatory inefficiency [100, 101].

Management of PSPC in PTLD cases anchors on PR, which improves the cardiopulmonary exercise, dyspnea scores, and quality of life across heterogeneous PTLD phenotypes [61, 100]. Thus, PR programs should incorporate aerobic training (walking or cycling with interval progression), resistance training (upper and lower limb), breathing retraining (pursed‐lip breathing, diaphragmatic strategies), and energy conservation techniques [61, 100]. In progressive fibrotic trajectories evidenced by serial declines in FVC/DLCO, worsening radiologic fibrosis, or symptomatic deterioration despite optimized supportive care, antifibrotic agents such as nintedanib should be considered along with careful monitoring for any adverse side effects [64, 66, 67]. In addition, long‐term oxygen therapy should be considered based on standardized assessment of resting and exertional hypoxemia [100]. Surgical interventions have a limited role in managing PSPC among PTLD cases but may be considered in carefully selected patients after multidisciplinary review, to remove localized and/or destroyed lungs that contribute to refractory infection or hemoptysis [12, 127].

Some of the caveats in the management of PSPC among PTLD cases includes: (a) Gas‐trapping and emphysema can coexist with fibrosis, which may create a pseudonormal spirometry result. To overcome this, the DLCO and HRCT pattern recognition may need to be coupled with spirometry [12, 100]. (b) Severe pleural disease can disproportionately impair lung function mechanics and may warrant targeted physiotherapy such as posture and stretching, and analgesic treatment to enable participation in PR measures [3, 19].

### 5.5 Chronic Pulmonary Aspergillosis

CPA is a major PTLD complication, particularly in patients with residual lung cavities. The predominant CPA pathogen is *Aspergillus fumigatus*, though *A. niger* and *A. flavus* can also be involved [3, 58, 61]. Individuals with CPA may display chronic cough, fatigue, weight loss, dyspnea, and hemoptysis [58, 61]. Since these symptoms overlap with PTLD, active surveillance of TB cases with cavities and constitutional symptoms, or persistent inflammatory markers should be implemented [58, 61]. Cavitary lesions predispose the lung to CPA, which though treatable, causes 20%–33% in short term, and 33%–80% mortality rates over a 5‐year period [3, 58, 61]. Diagnostic HRCT imaging of CPA cases reveals lung cavities with or without intracavitary fungal masses (aspergillomas), pericavitary fibrosis, or progressive pleuroparenchymal thickening [3, 58]. In addition, enzyme‐linked immunosorbent assay (ELISA)‐based serology testing for aspergillus‐specific IgG provides a more sensitive and specific CPA diagnosis, particularly when integrated with clinical and imaging tests [108, 128, 129, 130].

Treatment of CPA depends on the extent of lung damage and fungal infection and focused primarily on eradication of the etiological fungus through oral or inhaled antifungal agents [3, 61, 109]. The preferred first‐line therapy for CPA is oral itraconazole for 6–12 months, with therapeutic drug monitoring to ensure adequate exposure and minimize toxicity [61, 109]. As an alternative for intolerance, drug‐drug interactions, or resistance to itraconazole, voriconazole, isavuconazole, and posaconazole may be considered, while refractory disease may require intravenous amphotericin B or micafungin therapy [61, 109]. However, comanagement of drug and clinical improvement is crucial for effective therapeutic outcome [61, 107]. For example, the azole–rifamycin interactions should be monitored since rifamycin can dramatically reduce triazole levels. Similarly, hepatic function and QTc should be monitored, and wherever appropriate, hemoptysis management should be coordinated with bronchial artery embolization [61, 107]. Finally, not all aspergillomas require surgery, and the decision hinges on patient characteristics such as bleeding risk, cavity stability, and overall physiological reserve of the lungs [58, 109, 131]. While relapse of CPA is not uncommon, structured follow‐up and monitoring of cases using clinical, radiological and serological tools should determine the duration and adjustments of therapy, as needed [58, 109, 131].

### 5.6 PTLD in Nontuberculous Mycobacterial Diseases

Structural damage to lungs after TB predisposes to NTM pulmonary disease (NTM‐PD) through impaired mucociliary clearance, residual cavities, and altered local immunity. Epidemiological studies indicate that MAC (*Mycobacterium avium* complex) is the most common cause of NTM‐PD, followed by *M. kansasii* and *M. abscessus*, with prevalence estimates of ∼7%–11% among individuals with prior pulmonary TB in select cohorts [3, 61]. Similar to CAP, the clinical features of NTM‐PD, including chronic cough, sputum production, fatigue and weight loss overlaps with PTLD, and necessitate microbiologic confirmation to distinguish colonization from disease [59, 110, 111]. Diagnostic criteria include CXR, HRCT patterns (nodular‐bronchiecstatic vs. cavitary), bacteriological sputum smear/culture confirmation, and low to moderate complexity nucleic acid amplification technology (NAAT) as well as histopathology (Table 2). However, the specificity of disease diagnosis is strengthened by results from multiple positive cultures that are consistent and compatible with corresponding imaging results [59, 110, 111].

Therapy for NTM‐PD depends on the NTM species involved and prolonged. For *M. avium* complex‐pulmonary diseases (MAC‐PDs), regimens typically include rifampicin and ethambutol with a macrolide (clarithromycin or azithromycin) depending on macrolide susceptibility and disease pattern, while *M. kansasii* disease is treated with a combination of rifampicin, isoniazid and ethambutol [59, 111]. However, treatment of *M. abscessus*‐PD is challenging, often requiring multidrug regimens with parenteral agents tailored to subspecies, taking into consideration the inducible macrolide resistance. Importantly, the adverse toxicity and drug–drug interactions of these drugs necessitate close monitoring [110]. Apart from antibiotics, adjunctive airway clearance, vaccination, and PR measures can improve outcomes and reduce exacerbations of NTM‐PD in PTLD [12, 61]. Similar to the CPA, whenever NTM‐PD is complicated by bronchiectasis, integrating exacerbation prevention, such as consideration of inhaled antibiotics for bacterial cocolonization, helps to break the cycle of inflammation and improve treatment outcomes [12, 61].

Some of the key considerations for the management of NTM‐PD in PTLD includes: (a) In patients with persistent sputum symptoms and upper lobe bronchiectasis after TB, screening for NTM should be routine, particularly before escalating into a long‐term ICS, which may raise risk of mycobacterial infection and other chronic airway diseases; (b) Microbiologic clearance can lag symptom improvement; thus, maintaining therapy to guideline‐concordant durations after culture conversion is critical to reduce relapse of NTM disease [59, 111].

## 6 Integrated Diagnostics and Management Principles Across PTLD Phenotypes

A key component for effective PTLD management is the fundamental cyclic concept that the defects in lung structure guide its function, and the function guides the nature of therapeutic intervention. Thus, the baseline evaluation of lung structure abnormalities in PTLD cases should include: (i) CXR and/or HRCT to map cavities, bronchiectasis, air‐trapping, fibrotic bands, and pleural disease; (ii) spirometry with DLCO to diagnose phenotype obstruction, restriction, and diffusion defects; (iii) oscillometry or plethysmography when spirometry is nondiagnostic but PTLD symptoms persist; and (iv) microbiology to exclude Mtb relapse as well as to identify CPA or NTM, especially in symptomatic patients with cavities or upper lobe bronchiectasis [3, 12, 58, 59, 61, 100, 104, 105].

PR is a cross‐phenotype pillar and should be offered early and iteratively to suspected PTLD cases. The benefits of PR accrue even when the spirometric improvements are modest because exercise reconditioning, dyspnea management, and self‐management training can reduce symptom burden in the lungs and enhance quality of life among PTLD patients [61, 100]. In addition, vaccination (e.g., influenza and pneumococcal) and risk reduction (e.g., smoking cessation, improved nutrition and avoidance of biomass smoke) have been shown to prevent disease exacerbations and preserve lung health [61, 132]. In case of progressive fibrosis, careful selection for antifibrotic therapy in specialist settings is reasonable, although transparent discussions about expected benefits, monitoring, and unknowns in PTLD should be mandated [64, 66, 67]. Moreover, for central airway lesions, timely referral for interventional bronchoscopy can prevent lung deconditioning and recurrent infections by restoring airway patency [102, 103]. Finally, clinicians should be cognizant to evaluate hemoptysis due to the convergence of bronchiectasis, CPA, and fragile neo vasculature. In such cases, developing a coordinated protocol with interventional radiology for bronchial artery embolization may save lives and preserve lung parenchyma [61, 107].

In addition to therapy, active follow‐up of treated cases should be in place for effective PTLD management. For symptomatic or high‐risk patients, such as those with lung cavitation, extensive fibrosis and MDR‐TB history, periodic pulmonary function tests (PFTs), oxygen assessment, and targeted HRCT should be monitored periodically to track trajectory [4, 58, 59]. Similarly, for those with CPA or NTM, microbiologic/serologic monitoring schedule that aligns with clinical milestones should be established [4, 58, 59]. The main goal of follow‐up should be to anticipate progressive improvement in the lung structure and function, and not merely document it, so that airway clearance, rehabilitation intensity, interventional bronchoscopy, antifungal or antimycobacterial therapy, oxygen, or antifibrotic therapy can be timed to the need rather than delayed until disability has entrenched in the patients.

### 6.1 Risk Factors and Stratification of PTLD Management

PTLD is known to occur commonly with a previous history of TB, particularly with abnormal lung functions, spirometry measurements, radiological abnormalities and persisting respiratory issues, though other potential factors have been identified (Table 4) [48, 50, 51, 133]. Systematic review and meta‐analysis studies suggest that multiple factors modulate the likelihood and severity of PTLD. Analyses across diverse settings consistently implicate older age, prior TB treatment episodes (especially MDR‐TB), smoking, alcohol use, initial smear positivity, HIV status and greater radiologic lesion burden as contributors to long‐term impairment in PTLD. Risk factors such as food and lifestyle changes, including malnutrition, smoking and alcohol use, are classified as modifiable risk factors. Other risk factors, including age, HIV status, prior TB treatment, smear positivity and radiologic lesions, are nonmodifiable. In both, the effect or outcome is measured as odds ratio with a 95% confidence interval. The heterogeneity across the studies included in the meta‐analysis was assessed by *I*‐squared (*I* ^2^) expressed in percentage, with a higher percentage indicating greater variability [48, 50, 51, 133]. Delayed diagnosis and treatment have been indicated to attribute to PTLD where the delay could typically be patient‐ or healthcare‐centric. Risk factors, such as symptoms, age, healthcare‐seeking behavior, healthcare infrastructure, implementation strategy of diagnosis, shortage of testing kits, and reporting delays could result in a delay in diagnosis and treatment. The risk factor is hence modifiable or nonmodifiable depending on the individual reason for delay and cumulatively measured as delay time [41, 134].

**TABLE 4:** Risk factors for PTLD.

Risk factor	Effect size/association	Percentage of variability	Clinical implication for prevention/management	References
***Nonmodifiable***				
Older age	Odds ratio—1.62 (95% CI, 1.07–2.47)	*I* ^2^—77.9%	Consistent association with functional impairment	[48]
Prior TB episodes	Odds ratio—3.43 (95% CI, 2.37–4.97)	*I* ^2^—0.0%	Dose–response with impairment severity	[48]
Smear positivity	Odds ratio—3.11 (95% CI, 1.77–5.44)	*I* ^2^—0.0%	Marker of higher bacillary burden and tissue injury	[48]
Pulmonary lesions	Odds ratio—2.04 (95% CI, 1.07–3.87)	*I* ^2^—91.8%	Preexisting structural impairment	[48]
HIV (low CD4)	Prevalence ratio—32.79 (95% CI, 22.81–44.62)	*I* ^2^—82%	Mixed data; risk in specific contexts	[48, 50]
MDR‐TB	Odds ratio—3.27 (95% CI, 0.98–10.93)	*I* ^2^—0.0%	Mixed obstruction‐restriction, greater structural damage	[133]
***Modifiable***				
Smoking	Odds ratio—1.41 (95% CI, 1.09–1.83)	*I* ^2^—66.4%	Additive airway/parenchymal injury	[48]
Alcohol use	Odds ratio—1.84 (95% CI, 1.04–3.25)	*I* ^2^—0.0%	Additive airway/parenchymal injury	[48]
Malnutrition	Adjusted odds ratio—5.12 (95% CI, 1.36–19.33)	NA	Independent predictor of severe PTLD	[51]
***Mixed (modifiable and/or nonmodifiable)***				
Diagnostic delays	Delay days—1.79 (95% CI, 0.27–3.85)	*I* ^2^—99%	Increased severity and infectiousness	[134]
Treatment delays	Delay days—2.55 (95% CI, 0.54–4.56)	*I* ^2^—99%	Increased severity and infectiousness	[134]

Abbreviations: CD, cluster of differentiation; CI, confidence interval; HIV, human immunodeficiency; *I* ^2^, *I*‐squared; MDR‐TB, multidrug‐resistant TB; NA, not available.

Furthermore, both malnutrition and a history of respiratory failure emerge as independent predictors of severe PTLD in certain cohorts, highlighting the effect of systemic resilience on pulmonary recovery [51]. A previous study indicated that patients who were underweight carried 2.19 times more chances to develop PTLD compared to those with normal weight [135]. Undernutrition is often associated with poor immune functions, leading to unfavorable TB treatment outcomes and long‐term respiratory abnormalities which could lead to PTLD [105, 135].

### 6.2 Special Considerations for PTLD Diagnostics and Management

Overlap of various pulmonary ailment phenotypes is common and confounding in diagnosing PTLD cases. For example, patients may have upper lobe bronchiectasis from prior cavities, small‐airway disease from bronchiolitis, and traction bronchiectasis from fibrosis simultaneously [12, 58, 59, 61]. Therefore, PTLD management should prioritize the most limiting physiology (e.g., PR and oxygen in cases with severe diffusion impairment, airway clearance in exacerbation‐prone bronchiectasis, and interventional bronchoscopy for focal central stenosis), while addressing comorbid infection risk (e.g., CPA/NTM) and vaccination [12, 58, 59, 61]. It should be noted that the lungs of younger adults with PTLD may significantly be affected despite “mild” spirometry, reflecting depleted reserve [14]. Therefore, PR and proactive surveillance are equally relevant in this group as much as older and well‐established PTLD cases [14]. Importantly, when psychological distress and stigma accompany chronic symptoms or hemoptysis in PTLD cases, integrating psychosocial support should be considered to improve adherence to therapy to achieve positive outcomes [136].

### 6.3 Host Genetic Determinants of PTLD

Genetically determined immune responses shape both bacterial killing and irreversible pulmonary injury in PTLD. While microbiological cure eliminates Mtb, host genetic variants governing innate and adaptive immunity determine the magnitude, persistence, and resolution of inflammation, thereby dictating long‐term lung architecture. A central determinant lies in genes regulating intracellular mycobacterial control, notably *SLC11A1* (NRAMP1) gene. Polymorphisms in this gene affect phagolysosomal divalent cation transport and impair macrophage bactericidal capacity, thus prolonging intracellular persistence of Mtb, and sustaining antigenic stimulation [137, 138]. Prolonged macrophage activation amplifies TNF‐α, IL‐1β, and chemokine gradients, indirectly promoting matrix remodeling and fibrotic repair pathways that predispose to PTLD rather than efficient lesion resolution. Similarly, variants within the IL‐12–IFN‐γ axis modulate PTLD risk by setting thresholds for proinflammatory Th1 polarization and macrophage activation. Hypomorphic signaling in variants reduces sterilizing immunity and extends disease chronicity, whereas excessive or prolonged IFN‐γ signaling sustains granulomatous inflammation, hypoxia, and necrosis [139, 140]. These effects are compounded by delayed immune contraction, a critical determinant of PTLD.

The eicosanoid pathway, particularly polymorphisms in *LTA4H*, exemplifies genetically encoded inflammatory set‐points that directly tie immune phenotype to lung damage in TB [141, 142]. *LTA4H* promoter variants regulate the balance between proinflammatory leukotriene B4 and anti‐inflammatory lipoxins. Both hypo‐ and hyperinflammatory genotypes worsen TB outcomes, with hyperinflammatory states driving excessive neutrophil influx and bystander tissue damage [141, 142]. This bimodal effect underscores the concept that optimal, but not maximal, inflammation is required to minimize PTLD.

Expression of MMPs during TB is tightly linked to host genetics and inflammatory signaling. Elevated MMP‐1, MMP‐8, and MMP‐9 activity mediates collagen and elastin degradation, cavitation, and fibrotic remodeling [143, 144]. Hypoxia‐induced HIF‐1α signaling further amplifies MMP expression, establishing a feed‐forward loop between tissue hypoxia, inflammation, and matrix destruction that persists beyond Mtb clearance [145]. Finally, HLA Class I and II polymorphisms shape Mtb antigen presentation breadth and T‐cell effector profiles, influencing both immune efficiency and immunopathology of PTLD [146, 147]. Inefficient antigen presentation delays Mtb clearance, whereas overly robust cytotoxic responses exacerbate epithelial and endothelial injury, contributing to heterogeneous PTLD phenotypes [146, 147].

In summary, PTLD emerges as a genetically conditioned immunopathological sequela, where host gene variants that optimize early antimycobacterial defense paradoxically increase susceptibility to chronic lung injury when regulatory resolution pathways fail. However, as of 2026, there is no genetic variant reported specifically to PTLD. Most of the current PTLD literature, derived from transcriptomic and pathological data, emphasizes biological pathways, including MMPs, TGF‐beta, fibroblast activation, and tissue remodeling rather than reproducible inherited variants [148]. In addition, there is no evidence that the predominant genetic variant for TB‐severity (e.g., rs1848553 in RGS7BP, rs2976562 in SLA eQTL, plus immune‐response variants such as TLR2 rs5743708 and rs3804099) is also a PTLD variant [149, 150]. Therefore, although the immunopathologic and genetic characteristics of PTLD may appear to be distinct from, and much less well defined than, the genetics of TB severity, currently there are no PTLD‐specific genetic variants defined in humans.

## 7 PTLD in Children and Adolescents

### 7.1 Definition of PTLD in Children and Adolescents

PTLD in pediatric patients has increasingly been recognized as a consequence of childhood pulmonary TB. However, despite the large number of child TB survivors globally, PTLD remains understudied in this population compared to adult disease [151, 152]. The developmental context of PTLD in children is unique, since alveolarization, airway growth, and immune maturation continue through childhood and adolescence [151, 152]. Therefore, PTLD symptoms, such as parenchymal consolidation, atelectasis, or cavitary disease during childhood‐to‐adolescence stage can permanently reset lung growth trajectories and physiological reserve [151, 152]. Cross‐sectional and longitudinal epidemiological studies indicate that impaired spirometric measurement is common in children after months to years of anti‐TB treatment, more frequently marked with restriction, rather than obstruction [152, 153]. In addition, many children with PTLD show “apparently normal” spirometry despite persistent radiologic abnormalities, which implies latent loss of lung function and risk for future decompensation under respiratory stress [152, 153]. In a meta‐analysis of post‐TB lung function testing in children, the pooled effects indicated an adverse z‐score shifts in FEV_1_ and FVC (≈ −1.5 and −1.9, respectively), underscoring clinically meaningful deficits even when symptoms are modest [153].

### 7.2 Clinical Presentation of PTLD in Children and Adolescents

The radiological manifestations of pediatric PTLD range from fibrotic bands, calcifications, atelectasis, emphysema, and bronchiectasis with upper lobe predilection, mirroring the location of cavitary disease in older children and adolescents [151]. In these cases, bronchiectasis may be tractional (e.g., scar‐related) or infection‐driven, and its presence may correlate with chronic cough and recurrent exacerbations [151, 152]. Therefore, the PTLD management should emphasize careful differentiation of airway symptoms and measures to prevent exacerbation, prevention versus treatment for fibrosis, and so forth [151, 152]. Studies from several pediatric cohorts indicate that beyond the defects in lung structure, children and adolescents with PTLD had reduced health‐related quality of life (HRQoL) and persistent symptoms, such as wheezing and exertional dyspnea, after treatment completion, which is consistent with clinically relevant disability despite “cure” of TB [152]. These observations are supported by a recent cross‐sectional comparative study on pediatric TB cases, which showed a higher prevalence of lung function impairment and lower HRQoL among children postpulmonary TB versus peers without prior TB [154]. Analysis of studies from 875 children with a previous history of TB showed significantly reduced FEV1 and FVC1 ratio, though their FEV1/FVC1 ratio was preserved as compared to healthy controls. The data indicated a more restricted growth pattern rather than the severe obstructive pattern in children [155].

### 7.3 Factors Affecting PTLD in Children and Adolescents

It should be noted that the risk for PTLD among pediatric pulmonary TB cases is not uniform. In general, greater lung impairment has been shown to be associated with more extensive initial disease, presence of cavitary lesions, and delayed diagnosis and/or treatment, paralleling adult risk gradients [152]. In addition, nutritional status and age at disease onset can also impact the PTLD symptoms and severity [151, 152]. In a previous study with children (6–16 years) who completed TB treatment, to understand the effect of nutritional status, a BMI for age z‐score and previous history of TB were included in statistical analysis. The study indicated nutritional status having a strong association with lung functions, where the underweight population had poor alveolarization and slow lung growth resulting in abnormalities [156]. For example, malnutrition during TB may constrain lung growth and repair capacity; since children of younger age, particularly ≤ 5 years, are at a more vulnerable developmental stage of lungs, even short‐lived impairments can have long‐lasting consequences [151, 152, 156]. Poverty, malnutrition, and limited healthcare access can serve as challenging socioeconomic risk factors in children and adolescents with PTLD [157, 158]. HIV‐TB coinfection can also variably affect the risk of PTLD through synergistic immune and infectious burdens. However, epidemiological studies indicate heterogeneity in treatment access and baseline health of pediatric TB cases [152]. Thus, a context‐specific appraisal is necessary to properly evaluate the impact of HIV on PTLD in children [152]. Importantly, a significant evidence gap exists between various etiological agents and PTLD severity among preschool‐aged children, in whom standard spirometry is often infeasible and longitudinal data are sparse, highlighting the need for age‐appropriate measures and harmonized definitions [152]. Indeed, recommendations from pediatric PTLD working groups mirror these gaps and call for routine post‐treatment evaluation, especially in adolescents, who appear to have a high prevalence of abnormal spirometry and symptoms at follow‐up [136, 159].

### 7.4 Diagnosis of PTLD in Children and Adolescents

The diagnostic tools used to measure PTLD symptoms in children and the adolescent population should be tailored to their age. For school‐aged children and adolescents, spirometry with a reference z‐score may provide a pragmatic first line, while DLCO and plethysmography can add value when airway restriction or small airway disease is suspected, but spirometry is equivocal [152]. Similarly, in younger children, surrogates to spirometry, such as oscillometry, may be considered; however, the priority should be to standardize feasible follow‐up of these cases using symptom inventories, growth parameters, and periodic imaging, particularly when the risk is high (e.g., prior cavitation, extensive consolidation, or persistent symptoms) [151, 152].

### 7.5 Management of PTLD in Children and Adolescents

Although the management of PTLD is individualized for children, it shares several aspects with adult PTLD, and therefore, it should be adapted for growth and participation. For example, the PR activities should be tailored for age‐appropriate aerobic and strength activities, breathing retraining, and self‐management coaching, which can improve exertional tolerance and health status [152]. In cases presenting with airway disease and bronchiectasis, targeted airway clearance techniques and disease exacerbation prevention, including vaccination against influenza and pneumococcus, should be considered [152]. In adolescent PTLD cases with progressive fibrotic patterns, in which serial FVC/DLCO declines with concordant imaging, antifibrotic treatment strategies should be considered, though the evidence for the later intervention is limited and decisions should be made in multidisciplinary contexts [152]. Moreover, attention should be focused on any complications, such as CPA in those with pulmonary cavities and NTM disease in those with upper lobe bronchiectasis; in such cases, the diagnostic tool should be chosen to limit the identification of false positives while avoiding delay in implementing interventions [152].

Finally, equity and systems factors shape pediatric PTLD burden globally. For example, children in TB‐endemic countries face significant socioeconomical barriers to early diagnosis, imaging, and lung function testing, which increases the risk for morbidity and mortality due to PTLD in these subjects [151, 152]. To mitigate this, standardization of post‐TB follow‐up procedures, building pediatric‐friendly PFT capacity, and integrating nutrition support along with psychosocial care should be developed and implemented [151, 152]. Importantly, as the definitions of PTLD and standards for its diagnosis and treatment consolidate, embedding longitudinal pediatric PTLD surveillance into TB programs is vital to address the evidence gap and to ensure that cure translates into restored, durable lung health across childhood into adolescence and beyond.

## 8 PTLD Prevention and Health‐System Approaches

Prevention of PTLD should begin before TB treatment starts, by shortening time to diagnosis and initiating effective therapy promptly [49, 134, 160, 161, 162]. Patient‐ and system‐level delays, stemming from smear‐negative case presentations, coexisting chronic cough etiologies, sociobehavioral barriers, and limited access to diagnostics, are associated with more extensive disease and a higher risk of residual pulmonary impairment [49, 134, 160, 161, 162]. Because subclinical and percolator TB disease may contain metabolically active Mtb that are often transmissible, strategies that move beyond symptom‐based evaluation to active case finding (ACF) by scaling targeted chest radiography with bacteriologic confirmation can aid in early diagnosis along the spectrum and plausibly to limit downstream structural damage to the lungs [30, 31, 42]. After ruling out active TB, TB preventive treatment (TPT) for LTBI is a cornerstone of PTLD prevention because every averted case is averted structural injury to the lungs. For TPT, the RIF‐based short‐course regimens are preferred, than the longstanding adherence with isoniazid monotherapy that has toxicity issues [163, 164, 165]. Guidance derived from subsequent evidence endorses a 3‐month daily INH–RIF regimen (3HP) across age groups (≥ 2 years) and in people with HIV‐TB [163, 164, 165]. Furthermore, the 1‐month daily INH plus RIF regimen (1HP) was found to be noninferior to 9 months of INH monotherapy among people living with HIV (PL‐HIV) and had a higher completion rate and a similar safety profile, providing an additional practical option for scale‐up [163, 165, 166]. Key strategies to prevent PTLD through health‐system‐based approaches are discussed in the next section.

### 8.1 Intervention Points to Reduce PTLD

Interventions to reduce the intensity and duration of Mtb infection‐ induced tissue inflammation, early during treatment of active pulmonary TB, are likely to lessen structural sequelae in the lungs. Therefore, adjunctive host‐directed therapies (HDTs) proposed and tested in preclinical TB studies target pathways and networks implicated in tissue destruction and dysregulated repair [63, 69, 71, 72, 167]. Indeed, potential HDT agents, including doxycycline (as an MMP modulator), N‐acetylcysteine (NAC; to restore redox balance), vitamin D_3_, and autophagy inducers (e.g., rapamycin, phenylbutyrate) have promising and supportive clinical or translational evidence in TB and map directly onto the protease, oxidative stress, and immunometabolic circuits that drive PTLD [63, 69, 71, 72, 167]. A randomized, placebo‐controlled Phase II clinical trial to evaluate the adjunctive HDT potential of doxycycline for pulmonary TB demonstrated reduced levels of MMPs, ECM‐destruction markers, and decreased lung cavity volume. These observations strengthen the plausibility of using HDTs such as doxycycline to limit downstream sequelae of TB [63]. Similarly, early trials of NAC as a HDT for TB suggest a positive outcome in mitigating oxidative–stress‐related tissue injury and treatment toxicities, particularly in MDR‐TB, though additional and extensive studies are ongoing [167]. Previous studies, including nonhuman primates, indicated a role for fibrosis in TB granuloma, existing as a peripherally or centrally fibrotic structure, due to the heterogenous cytokine environment. Further, system biology indicated fibroblast to myofibroblast differentiation leading to fibrotic changes and the process could be therapeutically exploited to improve resolution of TB during treatment [168, 169]. Pirfenidone and Nintedanib, the most commonly recommended antifibrotic drugs, are proven to slow disease progression but not cure the damage [170, 171]. While used in the treatment of idiopathic pulmonary fibrosis patients, both drugs show an improved lung function and functional capacity of lungs [64, 172, 173]. In fact, the murine model established a role for Nintedanib in clearance, alleviating lung damage and reducing treatment time, with clinical trials underway establishing its role in HDT for TB [174]. Therefore, adjunctive HDTs should programmatically be embedded within the standard of care antimicrobial regimen, with relevant pharmacovigilance and drug–drug interaction monitoring, particularly for targeted pulmonary TB cases, such as those with high radiologic burden, cavitation, and high systemic inflammatory tone.

### 8.2 Health‐System‐Based Prevention of PTLD

The health‐system prevention efforts should ensure TB survivorship care after bacteriologic cure through antibiotic treatment. For example, PR, which includes structured aerobic and resistance training, and breathing retraining, can improve exercise capacity, dyspnea, and health status across heterogeneous PTLD phenotypes and should be offered early, not as a last resort to TB patients [61, 100]. Emerging multicenter data report gains in FEV_1_, FVC, DLCO, and 6‐min walk distance after PR, while nonrehabilitated comparators show functional decline over the same interval. Moreover, telerehabilitation and home‐based models may expand access where center‐based programs are scarce. Thus, embedding PR as a default post‐TB intervention pathway, for example, at treatment completion with follow‐up at 6 and 12 months, can operationalize prevention of disability, creates touchpoints for detecting complications (e.g., CPA, NTM), and normalizes long‐term lung health monitoring within TB programs [61, 100]. The WHO have released its first policy brief on long‐term disabilities during and after the completion of TB treatment and laid emphasis especially on PR [175]. A recent study highlighted the need for more flexible, individualized rehabilitation, which could be feasible under the local setting. The study emphasized the need to implement PR as part of national and global TB strategies beyond routine diagnosis and treatment [27, 176]. Studies with TB survivors with PTLD where PR typically included endurance training, strength training, breathing exercises, and airway clearance techniques along with nutritional and psychological support are provided. The PR showed good improvement in alleviating symptoms of PTLD as indicated by improvements in exercise tolerance, PFT, arterial blood gas analysis, 6‐min walk test, and muscle strength training. St. George's Respiratory Questionnaire (SGRQ), BMI, and generalized anxiety disorder (GAD) [19, 27, 177, 178]. These findings strengthen the recommendation for PR in the national TB program (NTP) as a long‐term health and patient wellbeing routine within the TB care framework. A typical 6–8‐week exercise program augmented with nutritional support and health counseling for adults; PR as a defined clinical standard in the assessment and management of PTLD; development of additional model for PR in children and adolescents; and inclusion of psychosocial support toward mental health [26, 27, 158, 176]. There are several ongoing clinical trials focused on risk factors, diagnosis, and management with the insight of multidisciplinary evaluation of pulmonary function and intervention through PR for improvement in treatment outcomes of PTLD (Table 5).

**TABLE 5:** Ongoing clinical trials of PTLD ‐ risk factors, diagnosis, and management^a^.

NCT number	Study title/objective	Sponsor	Time period	Age	Number of persons	Study type	Status	Outcome
NCT06047795	Endurance Training in Patients with Post‐TB Lung Disease	Riphah International University	3–6 weeks	18–50 years	36	Interventional	Completed	Results not yet posted
NCT07067528	The Feasibility and Acceptability of a Post‐Tuberculosis Lung Disease Diagnostic Algorithm in Uganda.	Mbarara University of Science and Technology	6 months	18 years and older	80	Interventional	Not yet recruited	Results not yet posted
NCT07178860	Respiratory Health and Wellbeing Postpulmonary Tuberculosis	Liverpool School of Tropical Medicine	1 year	18 years and older	50	Observational	Recruiting	Results not yet posted
NCT06744179	Post‐TB Sequelae Study	International Centre for Diarrhoeal Disease Research, Bangladesh	42 months	6 months and older	600	Observational	Recruiting	Results not yet posted
NCT06127641	Rehabilitation of People With Post‐Tuberculosis Lung Disease (HBPRP)	Centro Universitário Augusto Motta	4 years	18 years and older	40	Interventional	Completed	Improved exercise tolerance [179]
NCT06909799	A Confirmatory Trial of Adjunctive NAC to Prevent Post‐Tuberculosis Lung Disease	The Aurum Institute NPC	12 months	18–65 years	242	Interventional	Recruiting	Results not yet posted
NCT06597409	Respiratory Muscle Stretching for Improving Chest Expansion and Dyspnea in Post‐Tuberculosis Bronchiectasis	Universitas Padjadjaran	4 weeks	18–65 years	48	Interventional	Completed	Results not yet posted
NCT07097961	Paediatric Post‐TB Pulmonary Rehab Study	University of Iowa	6 weeks	6–17 years	40	Interventional	Recruiting	Results not yet posted
NCT07164742	Parent Study Name: Pulmonary Rehabilitation to Reduce Post‐Tuberculosis Morbidity (TB Pure)	Johns Hopkins University	48 weeks	18 years and older	690	Interventional	Recruiting	Results not yet posted
NCT07451899	TB Type and Spirometry Result vs. Functional Capacity Based on 6MWT in People with PTLD	Universitas Padjadjaran	12 weeks	10–18 years	20	Observational	Recruiting	Results not yet posted
NCT05426720	Risk Factors and Biomarkers for Post‐Tuberculosis Lung Damage	Peking University Third Hospital	3 years	25–60 years	400	Observational	Recruiting	Results not yet posted

Abbreviations: NAC, N‐acetylcysteine; PTLD, post‐TB lung disease; TB, tuberculosis; 6MWT, 6‐min walk test.

^a^ Data were sourced from clinical registration information available at the ClinicalTrials.gov website or from published literature (cited).

### 8.3 Management of PTLD‐Associated Health Manifestations

Preventing disease exacerbations and secondary infections can reduce the loss of lung function in structurally compromised TB survivors. For example, vaccination against influenza, pneumococcus, and SARS‐CoV‐2 is a low‐risk, high‐yield strategy to reduce the exacerbation frequency of severe lower respiratory tract complications in people with chronic lung disease, including PTLD [132, 180, 181]. Furthermore, vaccination coupled with other measures such as smoking cessation, improved household air quality (biomass exposure reduction), and nutrition support can stabilize symptoms and promote faster recovery of PTLD symptoms [48, 51]. In addition, regular health monitoring systems should maintain a low threshold evaluation of hemoptysis and overtreat relapsing infections cautiously, recognizing the frequent intersection of PTLD with CPA and NTM [58, 59, 107]. Particularly, the value of early embolization and NTM species‐directed therapy to avert catastrophic bleeding and recurrent, lung‐damaging exacerbations of CPA and/or NTM should be considered for effective PTLD management [58, 59, 107].

### 8.4 Implementation of Health‐System‐Based PTLD Management

Finally, the extent of implementation determines whether the prevention measures succeeded. Standardizing post‐TB follow‐up of high‐risk patients, such as those with extensive baseline disease, cavitation, MDR‐TB and/or delayed diagnosis, through symptom screen, spirometry with DLCO, targeted HRCT, and microbiology when indicated, may shift PTLD care from reactive to proactive [4]. Thus, integration of ACF with high‐quality linkage, short‐course TPT, targeted HDTs for severe inflammatory or cavitary disease, early PR, and routine vaccination, can form the backbone of a preventive PTLD pathway that can be adapted into current TB control programs with incremental investments [31, 42, 61, 100, 166, 182]. Some of the practical levers include one‐stop “TB survivor” visits at treatment completion, reflex referrals to PR, standing orders for vaccination, case‐manager‐led adherence support for TPT, and integrated radiology/physiology reporting that flags air‐trapping and fibrosis for tailored follow‐up for PTLD [31, 42, 61, 100, 166, 182]. Where feasible, registries and quality dashboards tracking PFTs, exacerbations, and hospitalization days can provide accountability and identify implementation gaps to address. The operational research for PTLD should also prioritize cost‐effectiveness and equity, ensuring these prevention packages reach the populations with the highest burden and least access to specialty care services. In sum, prevention of PTLD is achievable through earlier detection, short‐course prevention of progression, modulation of injurious inflammation, and structured survivorship care that treats TB not as an episode but as a condition with a preventable second act [31, 42, 61, 100, 166, 182].

## 9 Challenges and Knowledge Gaps in PTLD Management

Despite its growing recognition as a serious health concern, PTLD remains under‐represented in clinical practice and health policies of infectious disease management. Several cross‐cutting challenges, spanning from nosology and measurement, epidemiology and equity, to diagnostics, therapeutics and implementation science, limit the progress and point to concrete research and programmatic priorities for effective PTLD management.

### 9.1 Nosology, Phenotyping, and Clinical Measurement of PTLD

A first barrier is the lack of unified definitions for PTLD symptoms and decision‐making pathways for patient care, which hinders surveillance, clinical decision‐making, and trial comparability. Current dogma in the field underscores the need to consolidate PTLD as a distinct, post‐treatment condition of TB, with agreed structural/functional criteria and standardized reporting of obstructive, restrictive, and mixed phenotypes, alongside imaging correlates of PTLD [4, 12]. However, PTLD measures are inconsistent; for example, spirometry testing alone would miss small airway disease and diffusion defects. Therefore, routine inclusion of DLCO and, where feasible, oscillometry or plethysmography is required to unmask early impairment and to anchor longitudinal trajectories of PTLD [100, 104, 105]. Additionally, plethysmography and IOS can be used to differentiate obstructive from restrictive lung abnormalities. The role of IOS and body plethysmography in ILD, diffuse parenchymal lung diseases, lung hyperinflation, and airway obstruction has been well established [14, 115, 183, 184, 185]. IOS and body plethysmography of small and large airways, along with their resistance, reactance, impedance, and reactance area, are good predictors of lung abnormalities [184, 186, 187]. IOS with its improved precision and accuracy and the availability of portable IOS devices, PFT can be routinely used for assessment of the PFT in communities, especially in LMIC settings, offering effective screening for PTLD, especially among children, elderly, and critically ill patients, independent of patient effort [185, 188, 189, 190]. Imaging standards also need refinement, such as consensus in the lexicons and severity scoring for bronchiectasis, traction change, and pleural disease, which would bridge practice variation and enable structure/function modeling for lung function that can inform prognosis and clinical trial endpoints for potential PTLD therapy [3, 12]. Advances in assessment of PFT through different modalities like spirometry, IOS, body plethysmography, and imaging (CT and/or HRCT) and their implementation in the routine screening and diagnosis of lung abnormalities and function could impact clinical pertinence and therapeutic prognosis of PTLD.

### 9.2 Epidemiology, Equity, and External Validity of PTLD Symptoms

The epidemiologic database for PTLD remains skewed toward LMICs with limited representation from high‐income settings, creating questions of external validity and impeding global estimates of disability and economic burden [4]. The PTLD risk stratification consistently implicates older age, prior TB episodes, smear positivity, smoking, alcohol use, and radiologic lesion burden, with malnutrition and respiratory failure marking severe phenotypes in some cohorts; however, confounding from access to care and environmental exposures is not uniformly captured [48, 51]. Although the contribution of DR‐TB to mixed physiologic patterns in PTLD is obvious, the dose–response between treatment delays, disease kinetics, and irreversible lung injury remains underquantified across the TB spectrum, ranging from LTBI to cavitary disease [13, 49, 134, 160, 161, 162]. In addition, equity‐oriented PTLD management that incorporates air quality, biomass exposure, occupational risks, and nutrition is essential to convert risk associations into actionable, context‐specific prevention strategies [48, 51].

### 9.3 Diagnostics and Disease Complications

In classifying PTLD, the clinical course of action rarely embeds systematic microbiologic tests to detect CPA and NTM disease, which are frequent but treatable amplifiers of decline in structurally damaged lungs [58, 59]. Although current serologic and molecular tools for CPA and NTM have improved, the operational triggers, such as whom to test, when, and how often to test, are not standardized. Similarly, while interventional bronchoscopy can transform outcomes in tracheobronchial stenosis, referral thresholds and postprocedure follow‐up remain heterogeneous across different control programs [102, 103]. Therefore, a research priority is to test structured diagnostic bundles, including HRCT + spirometry/DLCO ± oscillometry + targeted microbiology, against usual standard of care, might be useful for earlier detection, fewer symptom exacerbations, and preserved lung function in PTLD subjects [12, 58, 59, 100].

### 9.4 Mechanisms and Biomarkers of PTLD

Research findings on PTLD indicate two mechanistically convergent axes, including the protease/MMP‐driven matrix destruction, TGF‐β/CTGF‐directed fibrotic responses, immunometabolic rewiring (HIF‐1α, succinate/itaconate), and NET‐associated epithelial injury; however, the clinical biomarkers that stratify PTLD risk or guide therapy are not yet validated for practice [12, 58, 59, 100]. At the mechanistic level, the evidence that supports MMP, TGF‐β, and CTGF as causal drivers of PTLD in humans is more nuanced than the evidence from preclinical studies. Though studies from human subjects support MMPs, TGF‐β, and CTGF as biologically plausible mediators associated with lung destruction and fibrosis, the degree of evidence for causality differs substantially. In general, human studies support a model in which MMPs, TGF‐β, and CTGF contribute to distinct but interconnected stages of PTLD. Among these mediators, MMPs, particularly MMP‐1, have the strongest evidence of causality [191, 192]. Elevated expression of MMPs in the areas of TB lesions with ECM destruction and cavitation correlates with disease severity and provides a biologically plausible mechanism for the irreversible tissue injury that precedes PTLD in humans [191, 192]. Similarly, TGF‐β has been reported to drive fibrotic repair response in humans [193]. Increased levels of TGF‐β were consistently associated with residual pulmonary fibrosis and pleural thickening after TB treatment, supporting its role in fibroblast activation, collagen deposition, and permanent lung remodeling [193]. In contrast, CTGF (CCN2) is best supported as a downstream profibrotic effector of TGF‐β signaling [194]. Studies have shown induction of CTGF and promotion of ECM production in human lung fibroblasts exposed to Mtb, although direct evidence linking CTGF to PTLD in human TB remains limited [194]. Collectively, the human data are consistent with a pathogenic cascade in which MMP‐mediated matrix destruction initiates lung injury, TGF‐β drives wound‐healing and fibrotic remodeling, and CTGF amplifies ECM deposition, ultimately leading to the structural abnormalities and functional impairment, which are characteristic of PTLD [191, 192, 193, 194].

Profiling of serum biomarkers for PTLD is recently gaining importance with the estimation of the inflammatory markers in patients with persistent lung damage following TB cure as predictors for post‐TB sequelae lung damage in addition to clinical, radiological, and pulmonary function assessment [195, 196, 197]. In previous studies on the systemic inflammatory markers and pulmonary functions, PTLD patients were compared with TB cured patients without sequelae, and a significant elevation of MMP‐1/TGF‐β was observed, indicating both as potential inflammatory biomarkers for PTLD [100, 193, 197, 198]. A 48‐panel Luminex multiplex assay was performed to understand the cytokines/chemokines and growth factors levels on post‐TB treatment individuals with minimal versus extensive lung damage. Among the biomarkers tested, levels of growth factors (basic fibroblast growth factor [FGF], hepatocyte growth factor [HGF], macrophage colony‐stimulating factor [M‐CSF], and stem cell growth factor‐beta [SCGF‐β]), proinflammatory cytokines (IL‐1α, IL‐2Rα, IL‐12p40, IL‐17, IL‐18, TNF‐α, and TRAIL), and anti‐inflammatory cytokine (IL‐4) were elevated in PTLD cases. Additionally, patients with extensive lung inflammation also showed upregulation of growth‐regulated oncogene alpha (Gro‐α) and monokine induced by gamma interferon (MIG) chemokines, as well as pleiotropic mediator, leukemia inhibitory factor (LIF). The sensitivities and specificities ranged from 47.06% to 73.53%, and 70.27% to 83.78%, respectively, indicating their diagnostic potential as biomarkers for persistent lung damage in conditions like PTLD [196]. Nonetheless, a multianalyte panel (e.g., MMPs/TIMPs, soluble TGF‐β/CTGF surrogates, metabolite fingerprints) and quantitative imaging platform can be built into prospective TB cohort studies to identify PTLD, and to develop progression and treatment–response scores, which is a prerequisite for precision clinical trials [15, 16, 100].

### 9.5 Potential Therapeutics and Clinical Trial Design for PTLD

Currently, PTLD management remains largely supportive, centered on PR, vaccination, and symptom phenotype‐directed pharmacotherapy, yet randomized evidence specific to PTLD is sparse [61, 100]. Although extrapolation of clinical care for other progressive fibrotic interstitial lung diseases supports the usage of nintedanib and pirfenidone in selected progressive fibrotic phenotypes, PTLD‐specific controlled clinical trials with clinically meaningful endpoints (e.g., FVC/DLCO decline, 6‐min walk, exacerbations, patient‐reported outcomes) are currently lacking [64, 66, 67]. Similarly, while HDTs, including doxycycline, vitamin D, rapamycin/phenylbutyrate, and NAC have mechanistic plausibility and early signals in TB, they need to be evaluated in PTLD‐oriented clinical trials [21, 199, 200]. These drugs can be administered to TB patients as an adjunct to anti‐TB treatment (to mitigate injury) and continued into early survivorship (to shape repair) with safety and microbiologic endpoints to avoid any trade‐offs [63, 69, 71, 72]. Thus, pragmatic designs, such as cluster or stepped‐wedge trials that embed PR plus airway clearance for bronchiectasis‐dominant phenotypes or antifibrotics for progression, can accelerate evidence generation while preserving internal validity for the utility of specific HDTs for effective PTLD management [61, 67].

### 9.6 Evidence Gaps in Pediatric and Adolescent PTLD

Meta‐analyses report of PTLD in children and adolescents showed adverse z‐score shifts in FEV_1_ and FVC and radiographic sequelae despite “normal” spirometry, implicating silent reserve loss of lungs and future vulnerability for PTLD [151, 152, 153]. However, age‐appropriate, repeatable physiology, including oscillometry where feasible, growth and symptom tracking, and periodic imaging for high‐risk profile cases, such as those with cavitation, extensive consolidation, severe disease at baseline, are yet to be operationalized into post‐TB clinics [151, 152]. Furthermore, the age bands and case definitions for PTLD in children and adolescents are not harmonized across various clinical studies or TB programs [151, 152]. Similarly, clinical trials tailored to youth PTLD management, such as PR intensity/frequency, airway clearance protocols, and school–house‐ or community‐based delivery of interventions, are overdue.

### 9.7 PTLD Prevention Across the TB Spectrum

Prevention of PTLD spans earlier detection of disease and prevention of secondary infections and/or lung damage after microbiological cure [30, 31, 42]. On the front end, ACF that addresses subclinical/incipient TB can reduce transmission and preempt lung injury [30, 31, 42]. After the completion of TB treatment, structured pathways should default to PR, vaccination (e.g., influenza, pneumococcus, SARS‐CoV‐2), smoking cessation, nutrition support, and scheduled physiology/imaging, while reserving targeted microbiology for high‐risk phenotypes and exacerbation‐prone patients [100, 112, 132, 180]. However, at present, most TB programs focus on bacteriologic cure as a treatment endpoint, leaving preventable disability, such as PTLD, unaddressed. Therefore, PTLD management should be integrated into current TB control programs worldwide.

### 9.8 Challenges in Implementing PTLD Care

A key gap in implementing PTLD care is how to deliver equitable, sustainable PTLD services. A prospective program should include clear referral thresholds (e.g., 6‐min walk < 80% predicted, mMRC ≥ 2, hemoptysis), task‐shared PR models, and linkage protocols to interventional bronchoscopy and CPA/NTM management [48, 51]. Similarly, the health‐economic analyses of PTLD should quantify productivity loss and cost savings from PR, vaccination, and early complication management to support investment in comprehensive TB control programs [48, 51]. In addition, PTLD implementation research can establish and test “TB‐survivor clinics” at treatment completion with a follow‐up at 6–12 months, comparing outcomes (e.g., exercise capacity, exacerbations and hospital admissions) versus usual discharge, and using quality dashboards (e.g., PFT coverage, PR uptake, vaccination rates) to collect data that would improve and refine PTLD management [61, 100].

In summary, the priorities to develop a consolidated PTLD management plan should include: (i) standardized PTLD definitions and measurement bundles; (ii) risk‐equity frameworks that integrate environmental and nutritional exposures; (iii) biomarkers and imaging metrics validated specifically for PTLD to predict disease progression and guide therapy; (iv) PTLD‐specific RCTs of antifibrotics, HDTs, and rehabilitation measures; (v) pediatric and adolescent surveillance and trials for PTLD; and (vi) implementation of an economic plan that embed survivorship care into current TB programs. Addressing these gaps in knowledge, infrastructure and implementation strategy can convert biological insight and clinical pragmatism of PTLD into measurable reductions in lung disability among TB survivors worldwide [4, 61].

## 10 Conclusions and Prospects

In conclusion, PTLD should be considered as a continuation of TB pathobiology, and not its epilogue. PTLD stems from TB‐induced persistent inflammation, protease‐mediated lung destruction, and profibrotic repair that imprint durable structural and functional deficits [15, 16]. The national policies and TB control program should focus beyond the diagnosis and treatment of active disease into the post‐TB sequelae and assessment of lung damage. In low‐resource high TB burden settings, where decentralized healthcare settings and lack of access to all diagnostic modalities like X‐ray or spirometry, symptom‐screening by simple symptom questionnaires is recommended [46, 162, 201, 202, 203]. In general, PTLD risk is amplified by older age, repeated TB, smear positivity, severe radiographic disease, malnutrition, and diagnostic/treatment delays, even though each of these can be an actionable prevention target [45, 48, 49, 51, 134]. Data from pediatric and adolescent TB cases reveal adverse spirometric z‐scores and persistent radiologic abnormalities in the lungs, despite “normal” results in clinical tests, underscoring silent reserve loss of lungs in these patients, and the imperative need for age‐appropriate follow‐up for potential PTLD development [151, 152, 153].

A practical, near‐term agenda for PTLD management should focus on interventions that are feasible within existing TB programs and target the major drivers of long‐term lung morbidity among TB survivors. First, standardized post‐TB lung health assessment should be routinely implemented after microbiologic cure. This includes spirometry and, where feasible, imaging to detect airflow obstruction, restriction, or mixed defects [20, 204, 205]. In patients with persistent symptoms or cavitary disease, microbiologic screening for CPA and NTM diseases is essential, as these complications are common, underdiagnosed, and treatable contributors to post‐TB morbidity [131, 206, 207, 208]. Second, routine PR and appropriate vaccination should be integrated into TB survivorship care. PR improves functional capacity, symptoms, and quality of life in chronic lung disease, while vaccination reduces secondary respiratory infections that can exacerbate structural lung damage [19, 27, 132, 209]. Third, patients with progressive fibrotic lung disease, identified by worsening symptoms, declining lung function, or serial imaging, should be triaged for antifibrotic evaluation and specialist referral [210, 211, 212]. Early identification of this subgroup is critical as PTLD can evolve into a progressive fibrosing phenotype. Fourth, PTLD prevention must address delayed TB diagnosis and modifiable risk factors, including smoking, indoor air pollution, recurrent TB, and comorbidities. Earlier detection and timely, effective TB treatment remain central to limiting irreversible lung injury [12, 49, 58, 59, 61].

Future priorities for effective PTLD management should include the development of molecular biomarkers to identify PTLD risk among TB‐survivors (MMP/TGF‐β/CTGF, immunometabolic signatures), PTLD‐specific randomized clinical trials to identify potential antifibrotics and HDT, harmonized surveillance of pediatric and adolescent TB cases for PTLD, and implementation of economic and policy plans that embed TB survivorship care within existing TB programs [4, 51, 67, 70]. Together, these steps can translate mechanistic insight into measurable reductions in chronic lung ailments due to PTLD among TB survivors worldwide.

## Author Contributions

**Radha Gopalaswamy and Selvakumar Subbian** conceived, drafted, and revised the manuscript. Both the authors have read and approved the final manuscript.

## Funding

The authors received no specific funding for this work.

## Ethics Statement

The authors have nothing to report.

## Conflicts of Interest

The authors declare no conflicts of interest.

## Data Availability

The authors have nothing to report.
